# Supplementary material for: Synthetic lethality of RB1 and aurora A is driven by stathmin-mediated disruption of microtubule dynamics
Source: Nat Commun. 2020 Oct 9;11:5105. doi: 10.1038/s41467-020-18872-0 (PMC7547687; doi:10.1038/s41467-020-18872-0)
Supplement: Supplementary file 1 — Supplementary Information [file 41467_2020_18872_MOESM1_ESM.pdf]

Supplementary Information

“Synthetic lethality of RB1 and aurora A is driven by stathmin-mediated disruption of microtubule dynamics”

Lyu et al.

(1) Supplementary Figures

(2) Full scans of Western blots

(3) FACS gating strategies

(4) Supplementary Tables

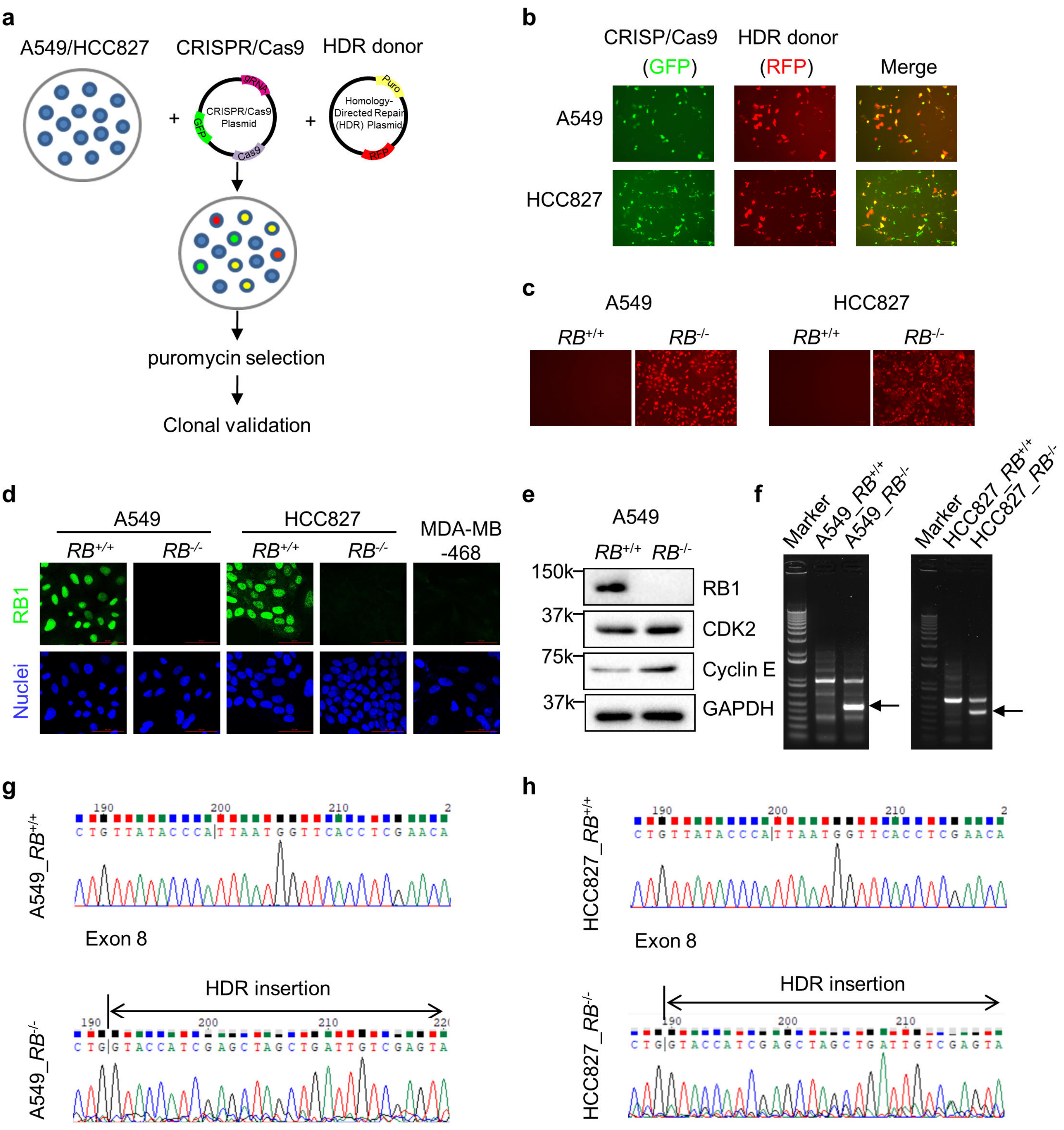

Supplementary Figure 1. Generation of RB1 knockout (KO) A549 and HCC827 lung cancer cells. a, Workflow of the generation of RB1 KO cell lines using CRISPR-Cas9 system. A549 or HCC827 cells were transfected with the CRISPR/Cas9 plasmid containing sgRNA and GFP marker and the HDR donor plasmid containing puromycin resistance and RFP markers. **b**, The transfection efficiency was assessed using GFP (CRSPR/Cas9 plasmid) and RFP (HDR donor plasmid). Scale bar = 100 μm. **c**, The representative image of the selected RB1 KO clones with red fluorescence. Scale bar = 100 μm. **d**, The RB1 KO clones were verified with immunofluorescence. Scale bars, 50 μm. **e**, Western blot analysis of CDK2 and cyclin E expression in A549 *RB1*$^{+/+}$ and A549 *RB1*$^{-/-}$ cells. **f**, PCR validation of the RB1 KO clones using primer pairs targeting Exon 8 of RB1 and loxP region in HDR plasmid, which was designed to amplify HDR insertion region of the RB1 locus. Exon 7 PCR did not show any HDR insertion (data not shown). **g** and **h**, Sanger sequencing analysis of the sgRNA target site on RB1 exon 8 in *RB1*$^{+/+}$ and *RB1*$^{-/-}$ cell lines.

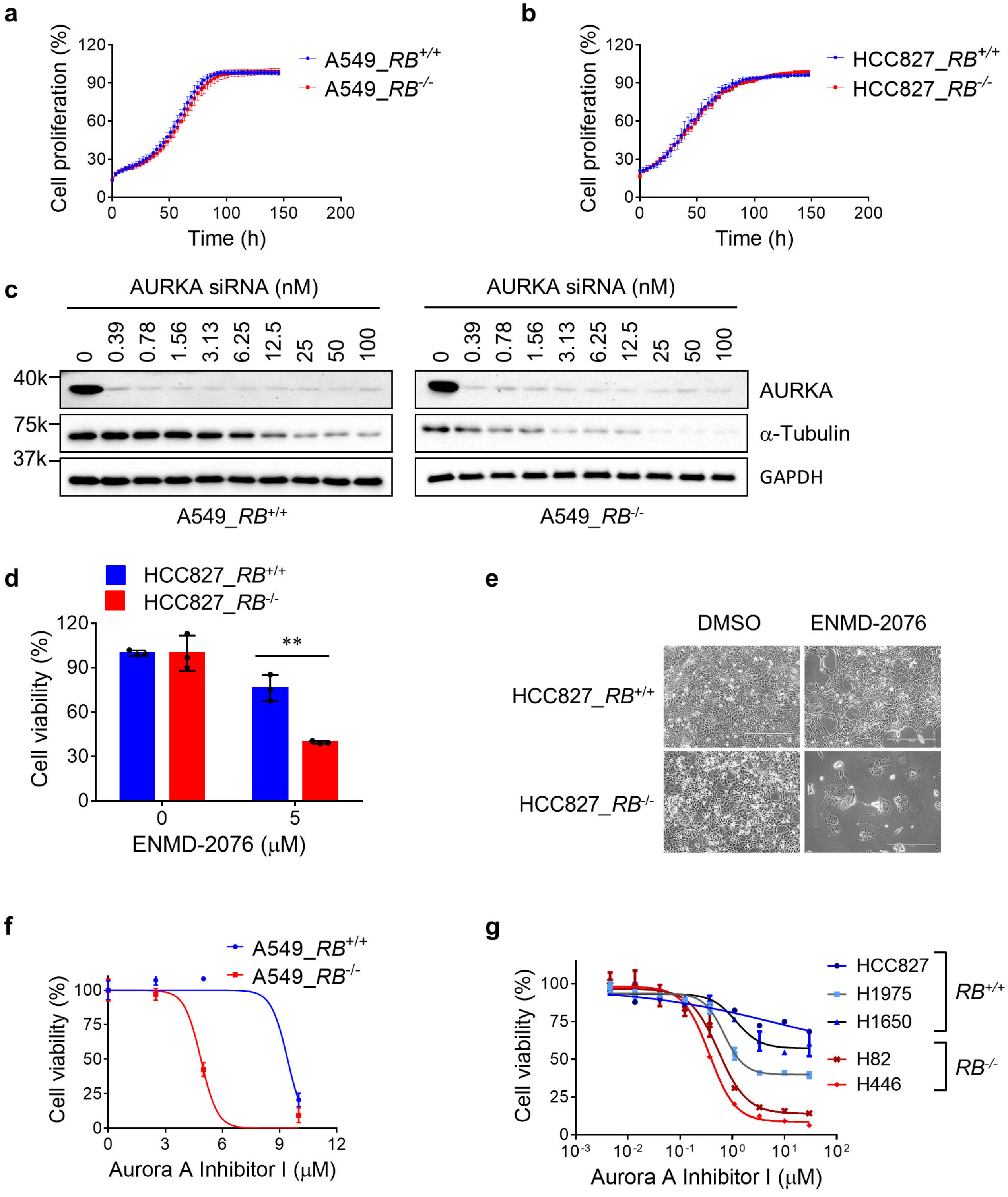

Supplementary Figure 2. Validation of the synthetic lethality between RB1 and AURKA. a and **b**, A549 (**a**) and HCC827 (**b**) RB1-isogenic cells were seeded in a 96-well plate at a density of 2,000 cells/well and incubated for 6 days. The cell proliferation rate was measured by real-time, live-cell imaging analysis with IncuCyte. Data are presented as mean ± SD (n = 3 independent experiments). **c**, A549 RB1-isogenic cells were treated with AURKA siRNA for 48 h and the levels of AURKA, α-tubulin and GAPDH were analyzed with Western blots. **d** and **e**, Effect of the AURKA inhibitor ENMD-2076 on the cell viability of RB1-isogenic HCC827 cell lines. HCC827 RB1 isogenic cells were treated with or without 5 μM ENMD-2076 for 72 h. Alamarblue cell viability data (**d**) and representative cell images (**e**) are shown. Scale bars, 400 μm. Data are mean ± SD of three independent experiments. **$P < 0.01$ between two groups. **f** and **g**, Effect of Aurora A Inhibitor I on RB1-isogenic A549 (**f**) and various lung cancer cell lines with different RB1 status (**g**). Cells were incubated with Aurora A Inhibitor I for 3 days and the cell viability was measured with Alamarblue staining. Data are presented as mean ± SD (n = 3 independent experiments).

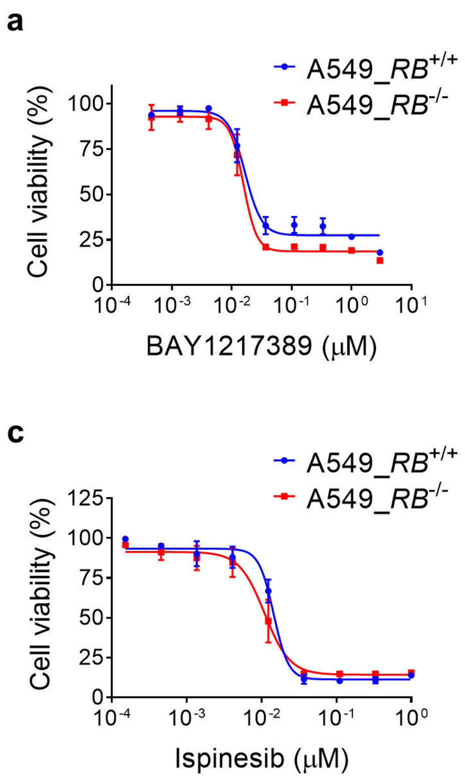

Supplementary Figure 3. Effect of general mitotic inhibitors on RB1-deficient lung cancer cells. A549 RB1-isogenic cells were incubated with BAY1217389, a TTK/Mps1 inhibitor (**a**), BI-2536, a PLK1 inhibitor (**b**), and Ispinesib, an Eg5 inhibitor (**c**), for 3 days and the cell viability was measured with Alamarblue staining. Data are presented as mean ± SD (n = 3 independent experiments).

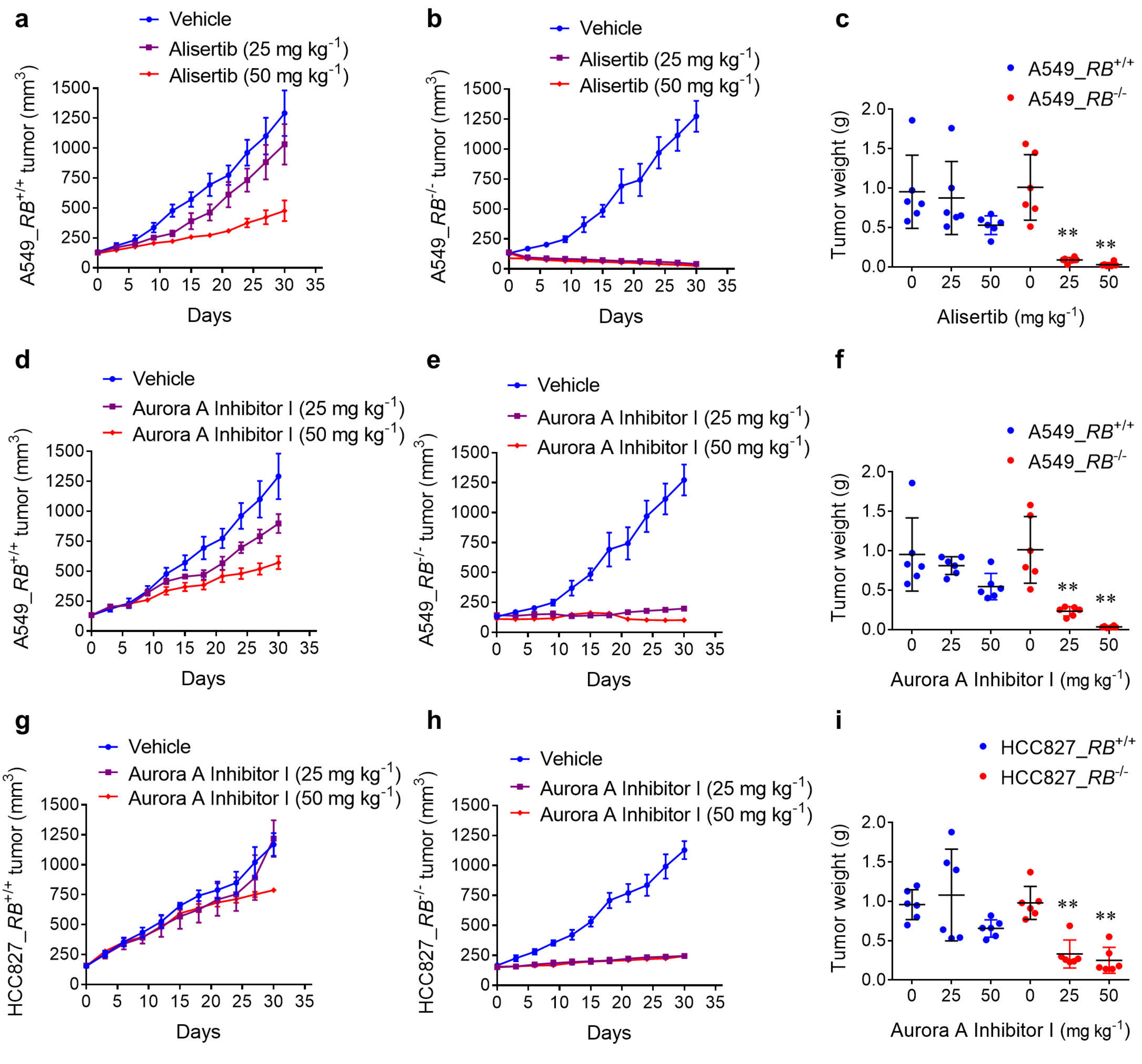

Supplementary Figure 4. RB1-isogenic lung cancer xenograft model. a-**c**, Effect of alisertib on A549 RB1-isogenic cell xenograft growth in mice. Tumor volume measurement (**a** and **b**) and tumor wet weight measurement (**c**) are shown. **d**-**f**, Effect of Aurora Inhibitor I on A549 RB1-isogenic cell xenograft growth in mice. Tumor volume measurement (**d** and **e**) and tumor wet weight measurement (**f**) are shown. Same control groups were used when A549 *RB1*$^{+/+}$ or *RB1*$^{-/-}$ tumor xenografts were tested for alisertib and Aurora A Inhibitor I. **g**-**i**, Effect of Aurora Inhibitor I on HCC827 RB1-isogenic cell xenograft growth in mice. Tumor volume measurement (**g** and **h**) and tumor wet weight measurement (**i**) are shown. Data are presented as mean ± SD (n = 6 independent animals per group). $^{**}P < 0.01$ vs no treatment control, determined using two-sided Student's t-test.

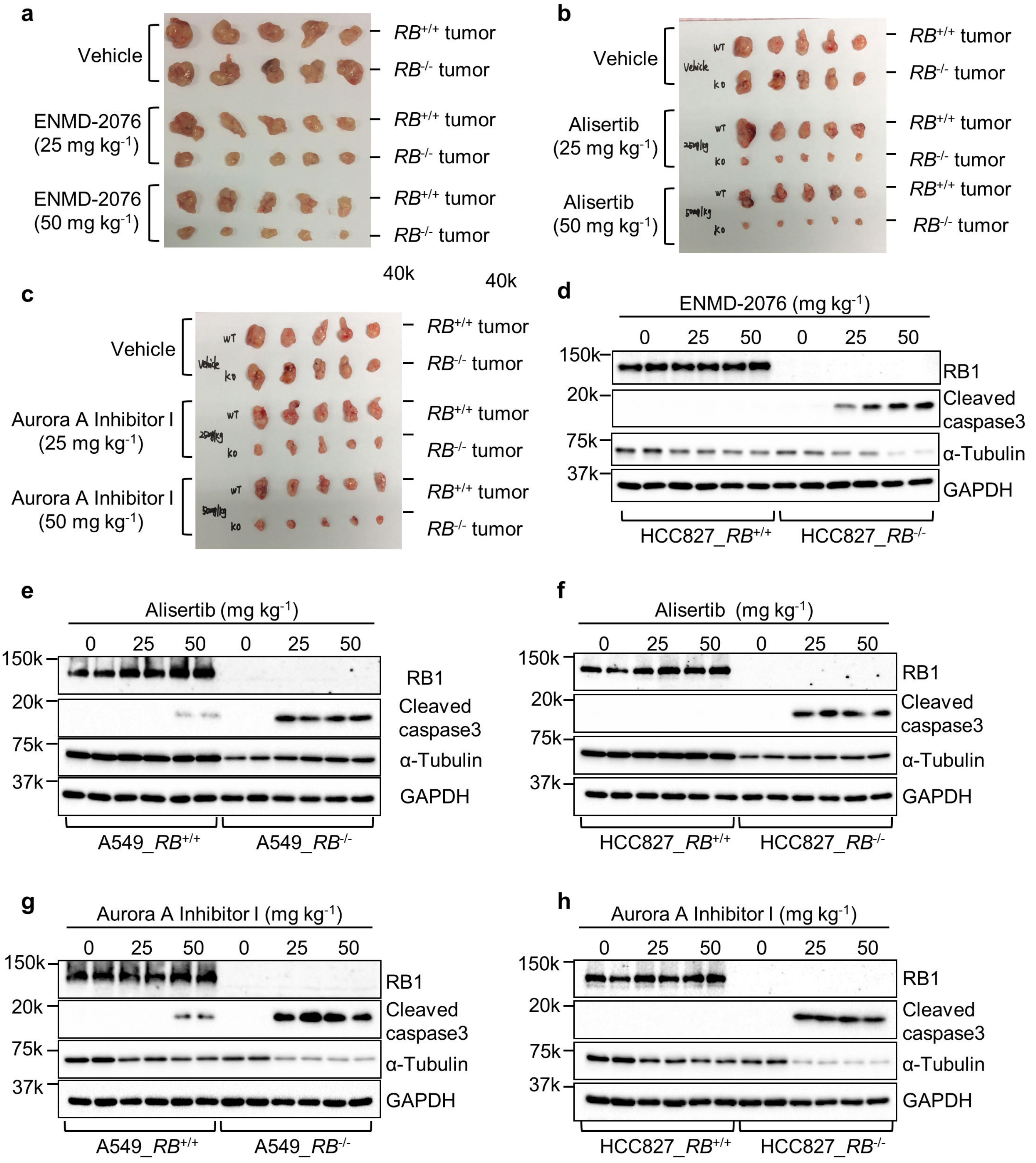

Supplementary Figure 5. RB1-isogenic lung cancer xenograft model. a-c, Representative images of tumor masses isolated from mice bearing A549 RB1-isogenic tumor xenografts treated with ENMD-2076 (**a**), alisertib (**b**) and Aurora A Inhibitor I (**c**). **d-h**, Western blots of proteins in tumor samples isolated from mice treated with ENMD-2076 (**d**), alisertib (**e** and **f**) and Aurora A Inhibitor I (**g** and **h**).

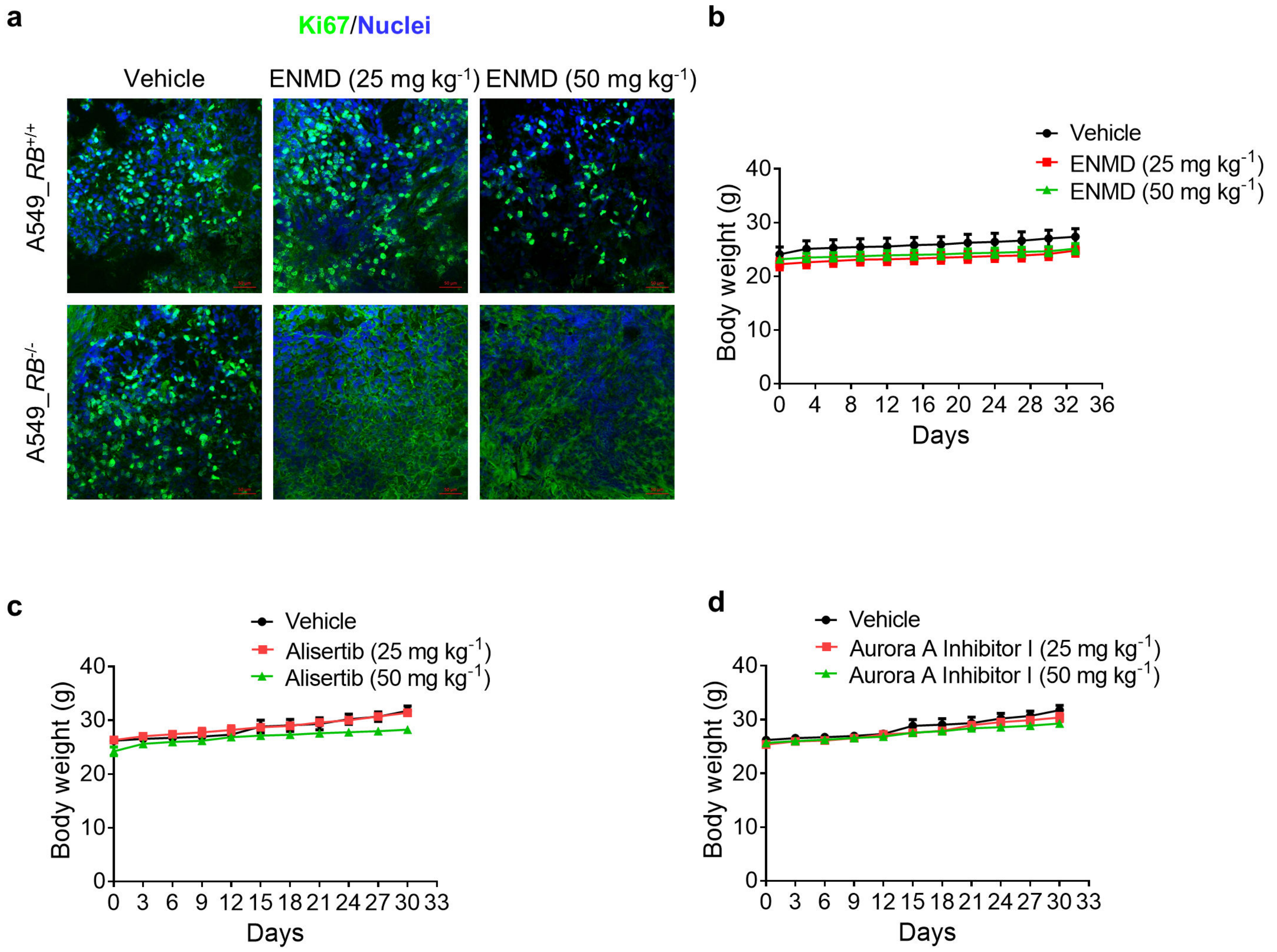

Supplementary Figure 6. RB1-isogenic lung cancer xenograft model. a Immunofluorescence staining of tumor tissue sections from the mouse xenografts using the cell proliferation marker Ki-67 (green) and the nuclear staining dye Hoechst33342 (blue). Scale bars, 50 μm. **b**-**d**, The measurement of mouse body weight during the treatment course of ENMD-2076 (**b**), alisertib (**c**), and Aurora A Inhibitor I (**d**) in A549 xenograft tumors in mice. Data are presented as mean ± SD (n = 6 independent animals per group).

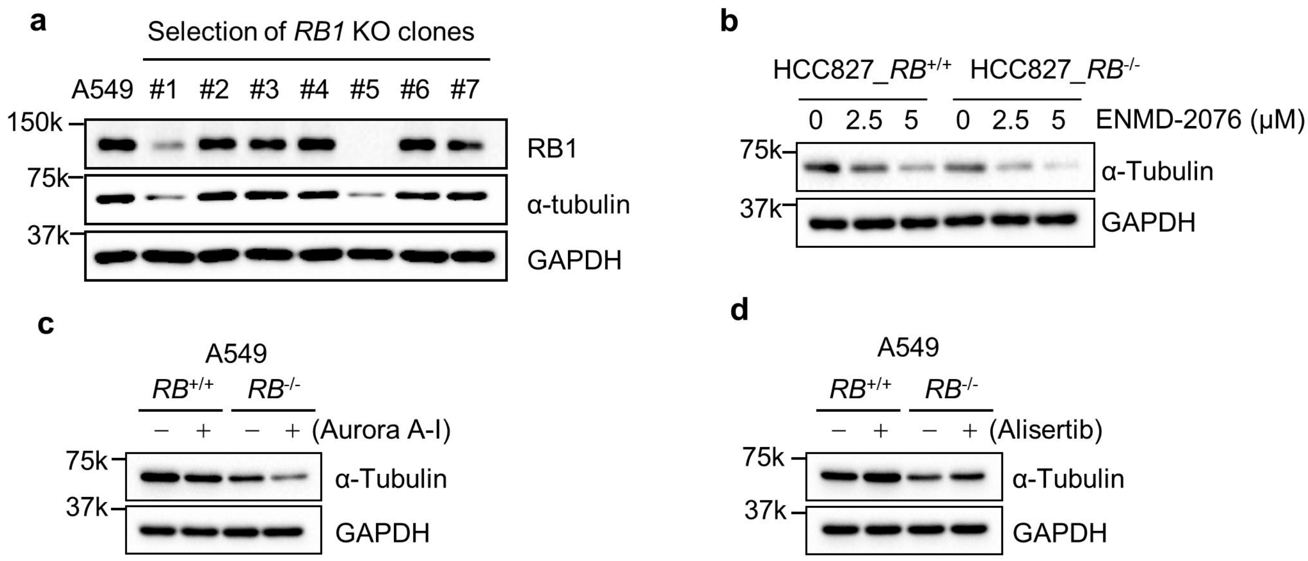

Supplementary Figure 7. Effect of RB1 KO and AURKA inhibition on α-tubulin level in RB1-isogenic cells. a, Comparison between RB1 and α-tubulin protein levels. Several A549 cell clones during the selection of RB1 CRISPR/Cas9 KO clones were analyzed for Western blots of RB1 and α-tubulin levels. **b**, Effect of ENMD-2076 on α-tubulin level in HCC827 RB1-isogenic cells. **c** and **d**, Effect of Aurora A Inhibitor I (Aurora A-I, 5 μM) and alisertib (10 μM) on α-tubulin level in A549 RB1-isogenic cells.

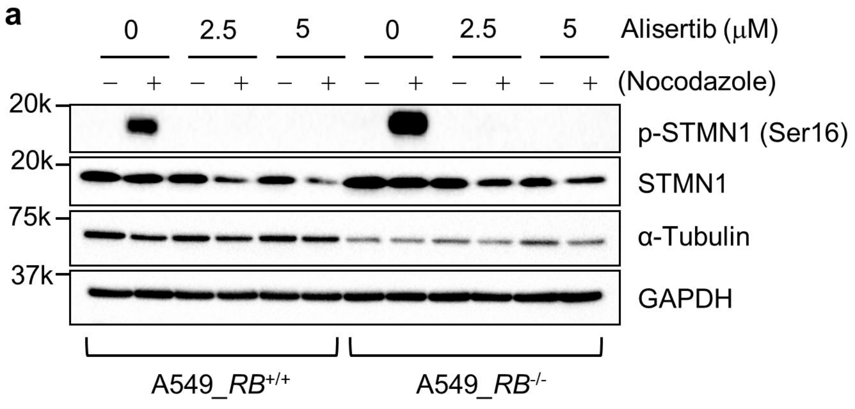

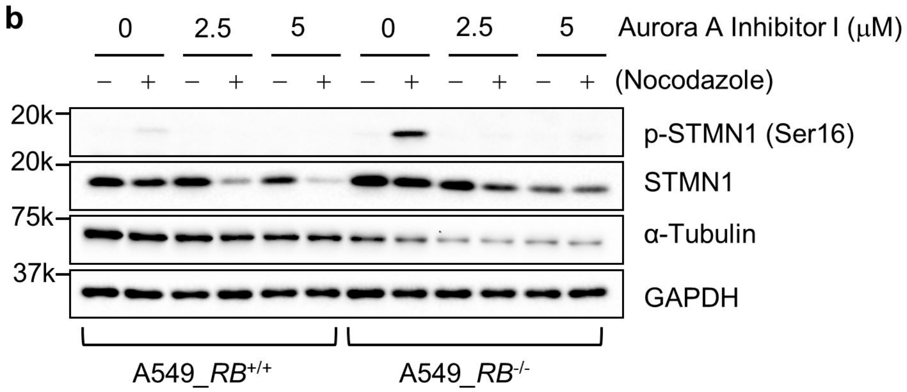

Supplementary Figure 8. Effect of AURKA inhibitors on stathmin phosphorylation. a and **b**, Effect of alisertib and Aurora A Inhibitor I on stathmin phosphorylation. Cells were treated with alisertib (**a**) or Aurora A Inhibitor I (**b**) for 24 h and then 100 nM nocodazole was treated for additional 24 h, prior to the Western blot analyses of phospho-stathmin at Ser16, total stathmin and α-tubulin.

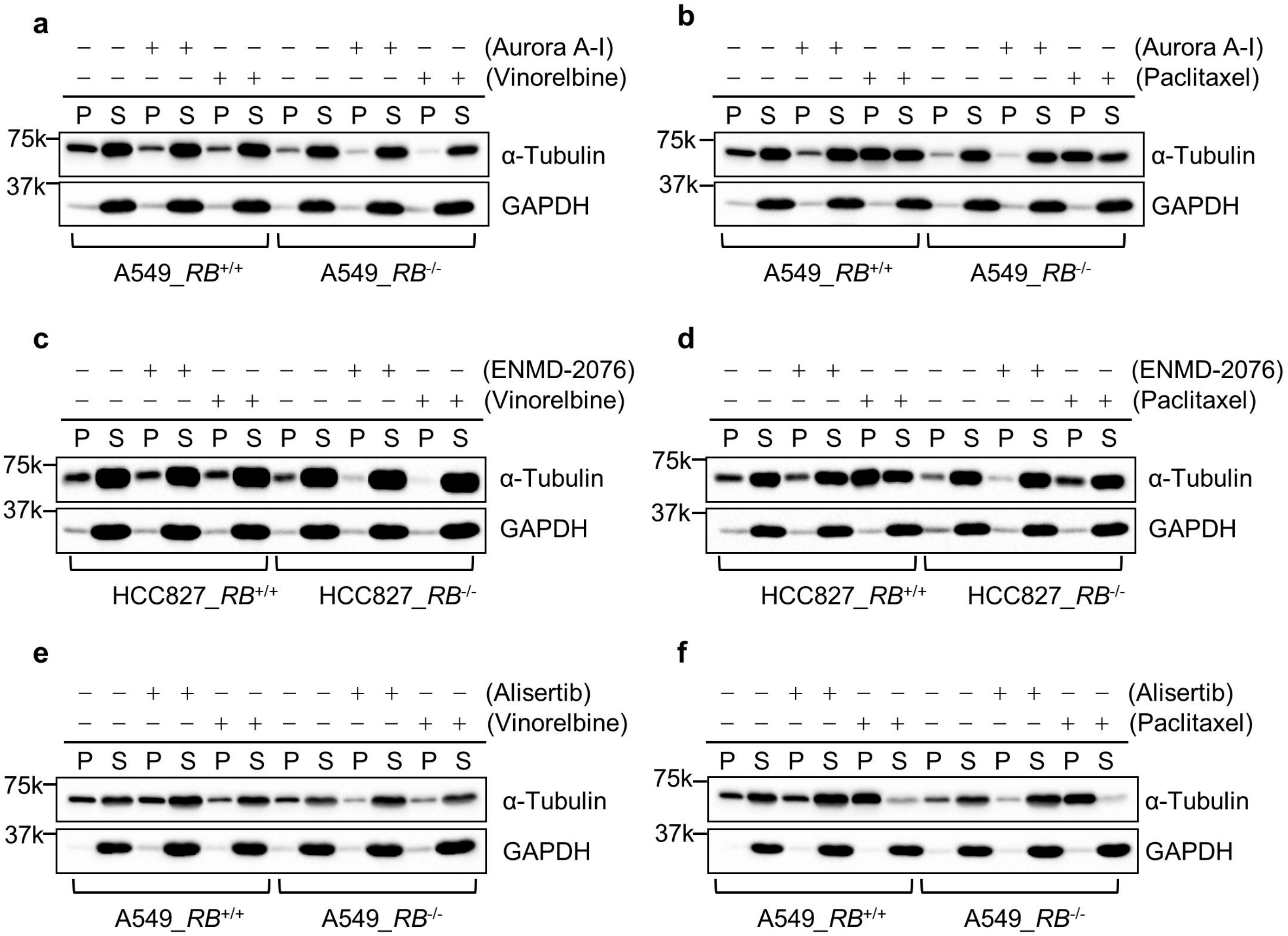

Supplementary Figure 9. Effect of AURKA inhibitors on microtubule polymerization in RB1-isogenic cells. **a**-**f**, Effect of AURKA inhibitors, vinorelbine (microtubule destabilizer) and paclitaxel (microtubule stabilizer) on polymeric microtubules. HCC827 or A549 RB1-isogenic cell pairs were treated with 5 μM Aurora A Inhibitor I (Aurora A-I) (**a** and **b**), 5 μM ENMD-2076 (**c** and **d**), 10 μM alisertib (**e** and **f**), 25 nM vinorelbine and 25 nM paclitaxel for 24 h and then the cells were fractionated into the soluble (S) and polymerized (P) microtubule fractions. The samples were subjected to Western blot with α-tubulin antibody. Vinorelbine and paclitaxel were tested in parallel as positive controls for microtubule destabilization and stabilization, respectively. GAPDH was used as a loading control.

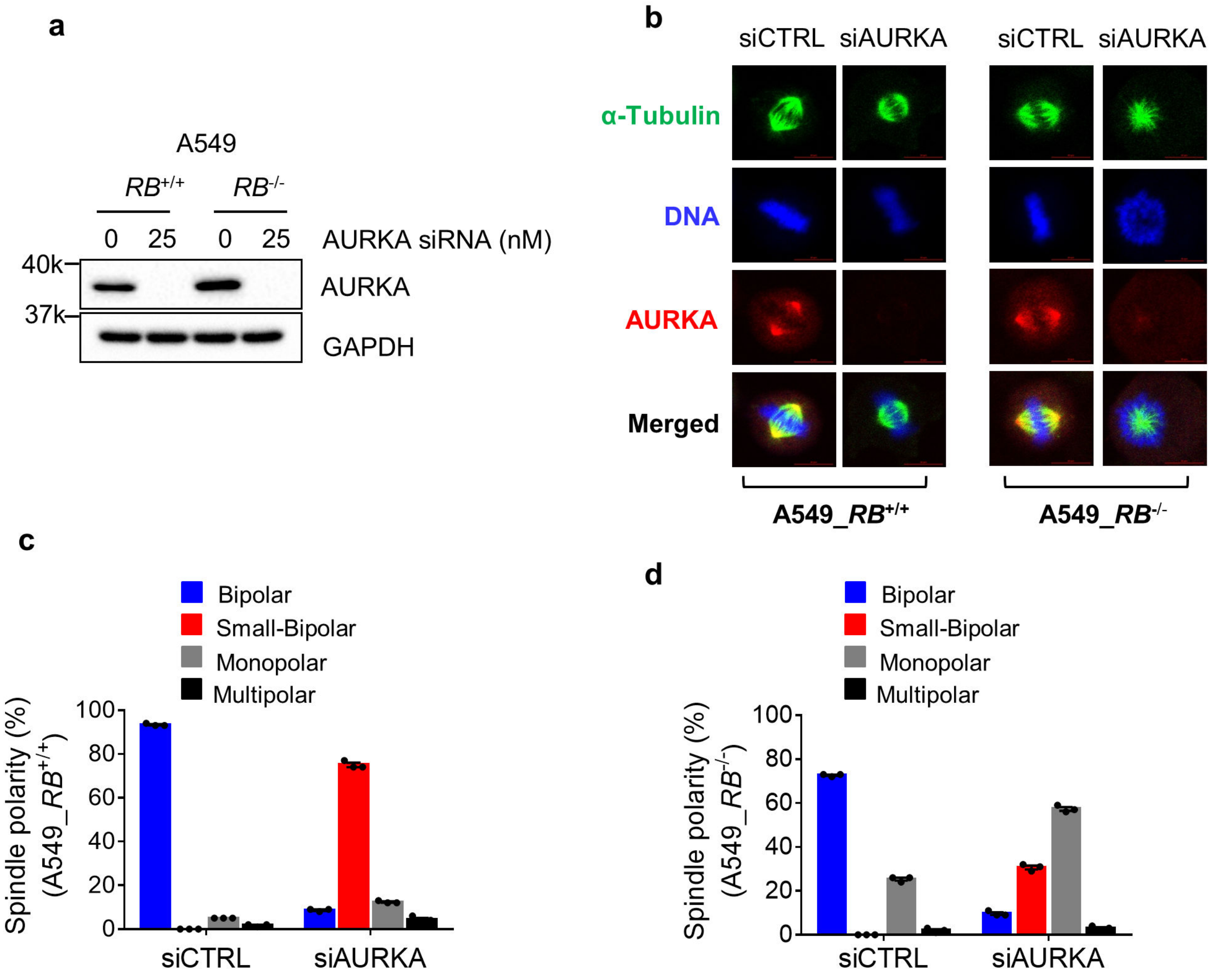

Supplementary Figure 10. Effect of AURKA silencing on spindle polarity in A549 RB1-isogenic cell pair. **a**-**d**, Spindle morphology analysis of mitotic cells treated with AURKA siRNA. A549 *RB1*$^{+/+}$ and *RB1*$^{-/-}$ cells were transfected with 25 nM AURKA siRNA (siAURKA) or control siRNA (siCTRL) for 48 h. Bortezomib (100 nM) was added at the last 2 h. **a**, Western blot analysis was done to verify silencing efficiency. **b**, The spindle and mitotic DNA were analyzed with the immunofluorescence staining of α-tubulin (green), AURKA (red) and DNA (blue). Scale bars, 10 µm. **c** and **d**, Mitotic cells from the confocal images were analyzed for spindle polarity and quantitated based on four criteria: normal bipolar, small (short) bipolar, monopolar and multipolar. Data are presented as mean ± SEM (n = 3 independent experiments. For each experiment, total 100 mitotic A549 *RB1*$^{+/+}$ cells (b) and total 100 mitotic A549 *RB1*$^{-/-}$ cells from each treatment condition were analyzed).

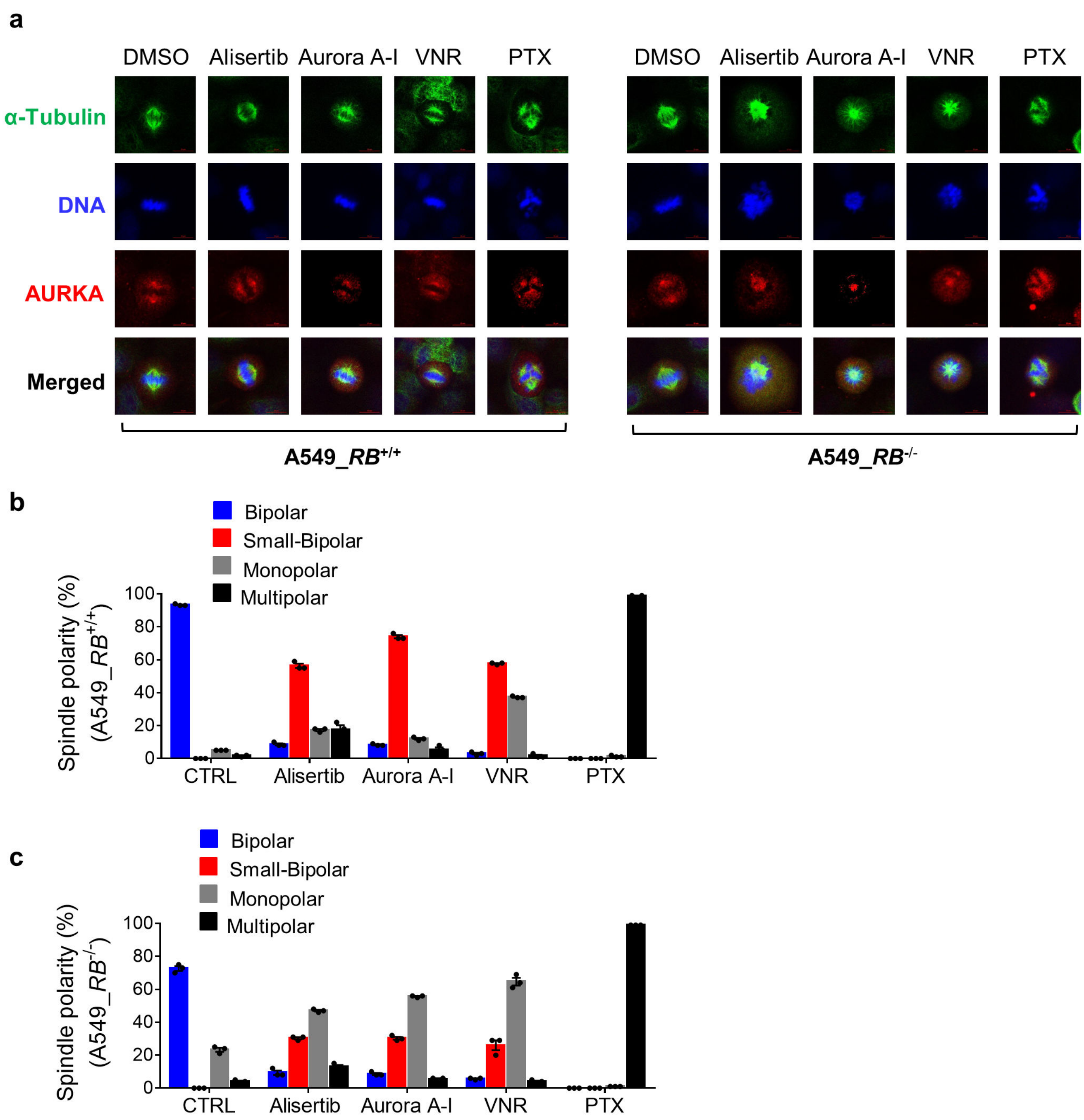

Supplementary Figure 11. Effect of AURKA inhibitors on spindle polarity in A549 RB1-isogenic cell pair. a-**c**, A549 *RB1*$^{+/+}$ and *RB1*$^{-/-}$ cells were treated with 2.5 μM alisertib, 2.5 μM Aurora A Inhibitor I (Aurora A-I), 25 nM vinorelbine (VNR) and 25 nM paclitaxel (PTX) for 24 h. Bortezomib (100 nM) was added at the last 2 h. **a**, The spindle and mitotic DNA were analyzed with the immunofluorescence staining of α-tubulin (green), AURKA (red) and DNA (Hoechst33342, blue). Scale bars, 10 μm. **b** and **c**, Mitotic cells from the confocal images were analyzed for spindle polarity and quantitated based on four criteria: normal bipolar, small (short) bipolar, monopolar and multipolar. Data are presented as mean ± SEM (n = 3 independent experiments. For each experiment, total 100 mitotic A549 *RB1*$^{+/+}$ cells (b) and total 100 mitotic A549 *RB1*$^{-/-}$ cells from each treatment condition were analyzed).

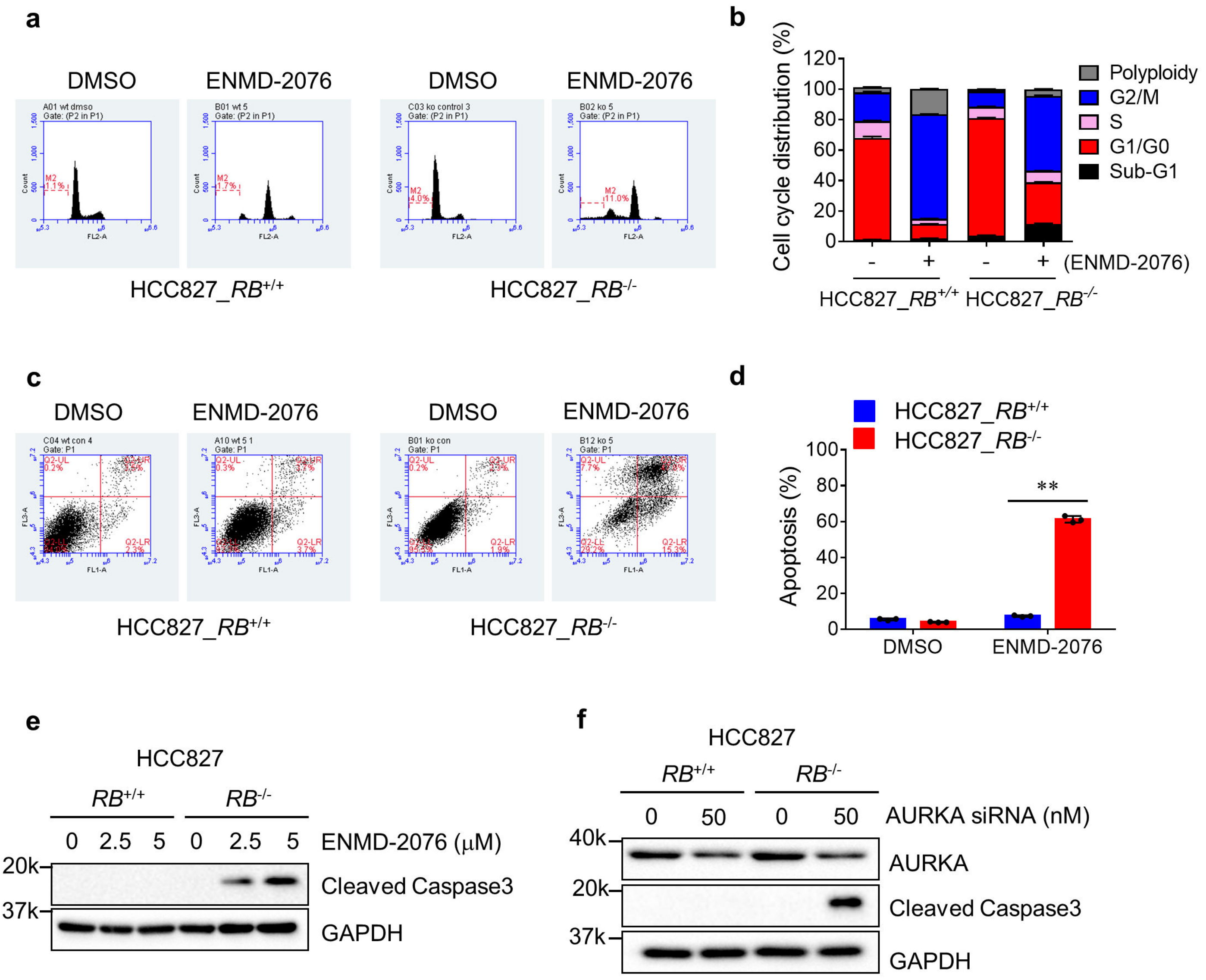

Supplementary Figure 12. Effect of AURKA inhibition on cell cycle and apoptosis in HCC827 RB1-isogenic cells. a and **b**, Effect of ENMD-2076 on the cell cycle progression of HCC827 *RB1*$^{+/+}$ and *RB1*$^{-/-}$ cells. Cells were treated with 5 µM ENMD-2076 for 48 h and the cell cycle was analyzed by flow cytometry. The cell cycle distribution was quantified (**b**). Data are presented as mean ± SEM (n = 3 independent experiments). **c** and **d**, Effect of AURKA inhibitor on apoptosis of HCC827 *RB1*$^{+/+}$ and *RB1*$^{-/-}$ cells. Cells were treated with 5 µM ENMD-2076 for 72 h and cell apoptosis was measured with Annexin V-FITC/propidium iodide staining. Data are presented as mean ± SEM (n = 3 independent experiments). ***P* < 0.01 between two indicated groups, determined using two-sided Student's t-test. ∗∗*P* < 0.01 between two groups. **e** and **f**, Effect of AURKA inhibition on apoptosis of HCC827 *RB1*$^{+/+}$ and *RB1*$^{-/-}$ Cells. Cells were treated with the indicated concentrations of ENMD-2076 (**e**) or AURKA siRNA (**f**) for 72 h and cell apoptosis was measured with the Western blots of cleaved caspase-3.

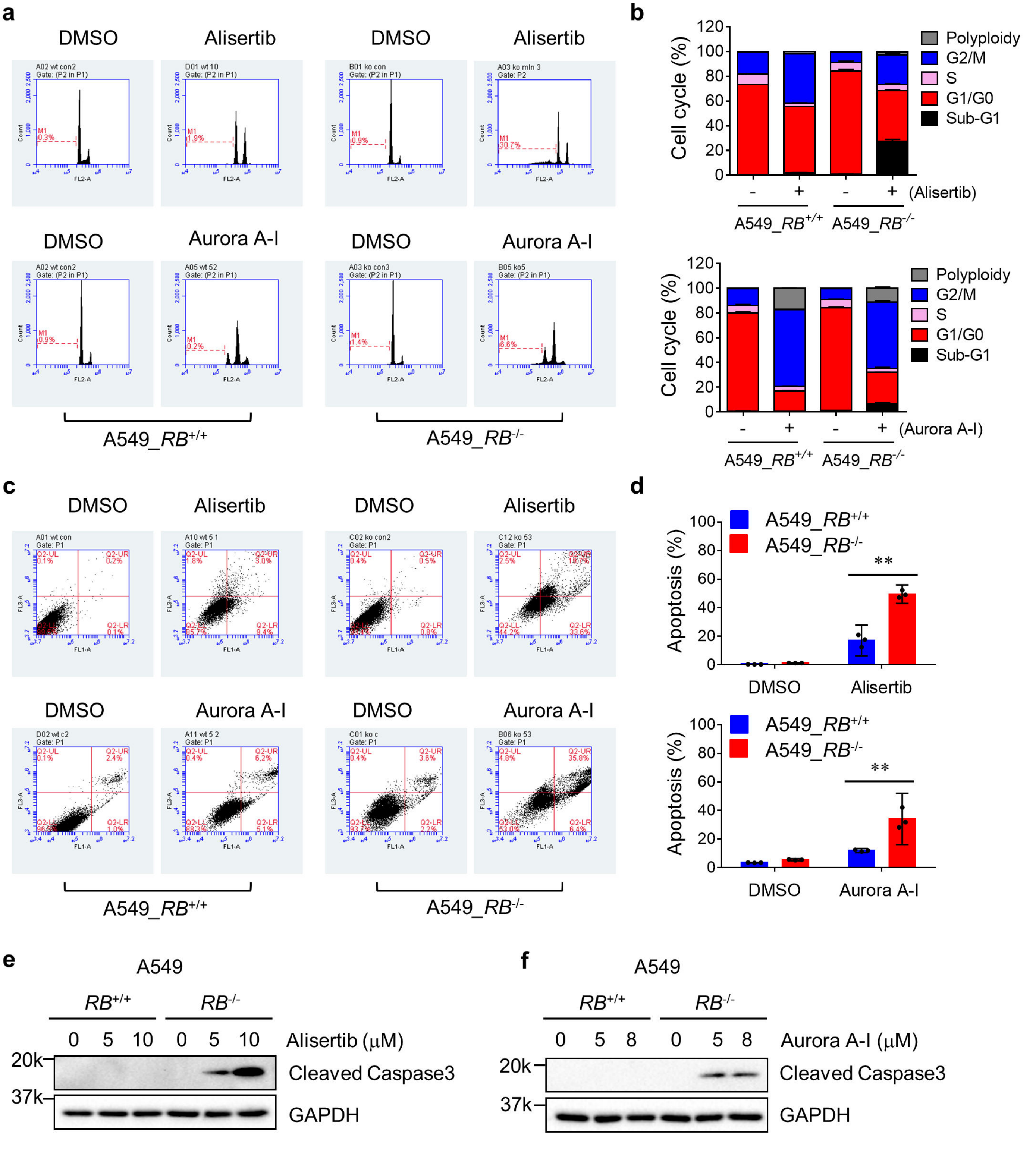

Supplementary Figure 13. Effect of AURKA inhibitors on cell cycle and apoptosis in A549 RB1-isogenic cells. a and **b**, Effect of alisertib and Aurora A Inhibitor I (Aurora A-I) on the cell cycle progression of A549 *RB1*$^{+/+}$ and *RB1*$^{-/-}$ cells. Cells were treated with 5 μM alisertib and 5 μM Aurora A Inhibitor I for 72 h and the cell cycle was analyzed by flow cytometry. The cell cycle distribution was quantitated (**b**). Data are presented as mean ± SEM (n = 3 independent experiments). **c** and **d**, Effect of alisertib and Aurora A Inhibitor I (Aurora A-I) on apoptosis of A549 *RB1*$^{+/+}$ and *RB1*$^{-/-}$ cells. Cells were treated with 5 μM alisertib and 5 μM Aurora A Inhibitor I for 72 h and cell apoptosis was measured with Annexin V-FITC/propidium iodide staining. **d**, Data are mean ± SD of three independent experiments. Data are presented as mean ± SEM (n = 3 independent experiments). ***P* < 0.01 between two indicated groups, determined using two-sided Student's t-test. **e** and **f**, Measurement of caspase-3 cleavage in A549 RB1-isogenic cells treated with alisertib or Aurora A Inhibitor I for 72 h.

Figure 1a

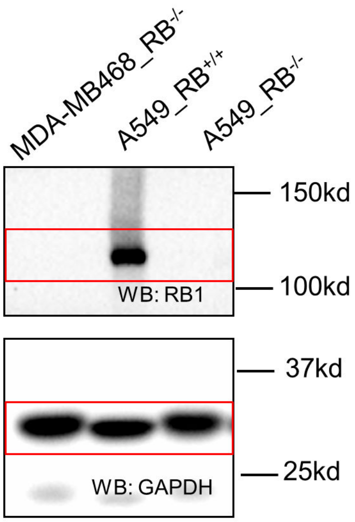

Figure 1b

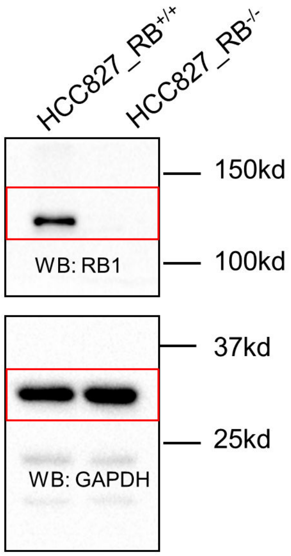

Figure 2k

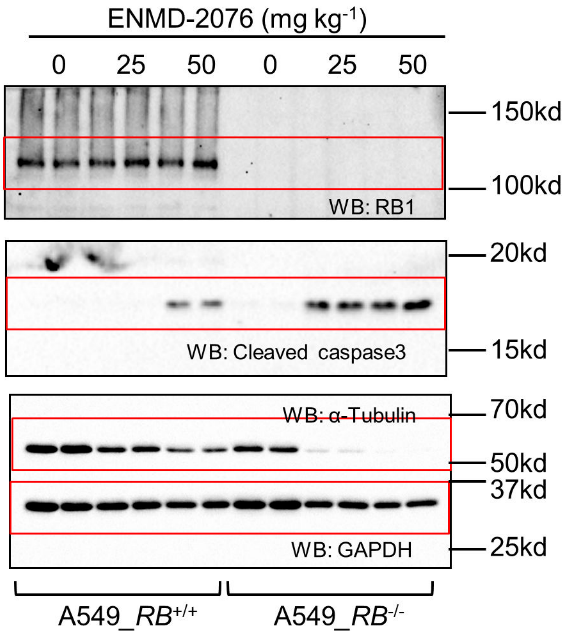

Supplementary Figure 14. Original Western blots shown in Figures 1-2. Each figure corresponds to the Western blots in the indicated Figure number.

Figure 3a

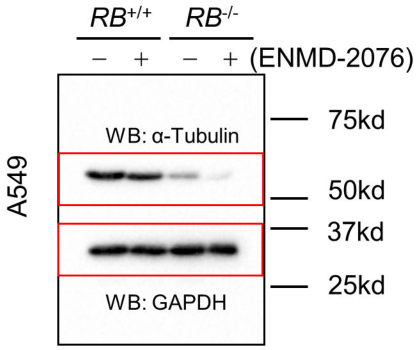

Figure 3b

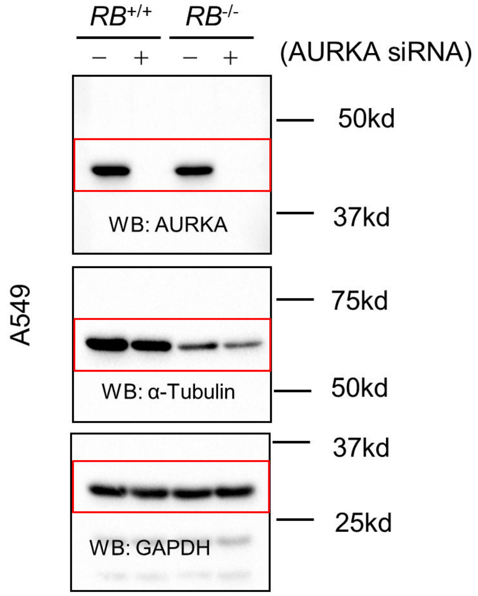

Figure 3c

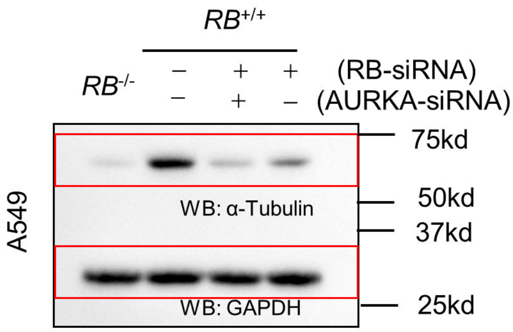

Figure 3e

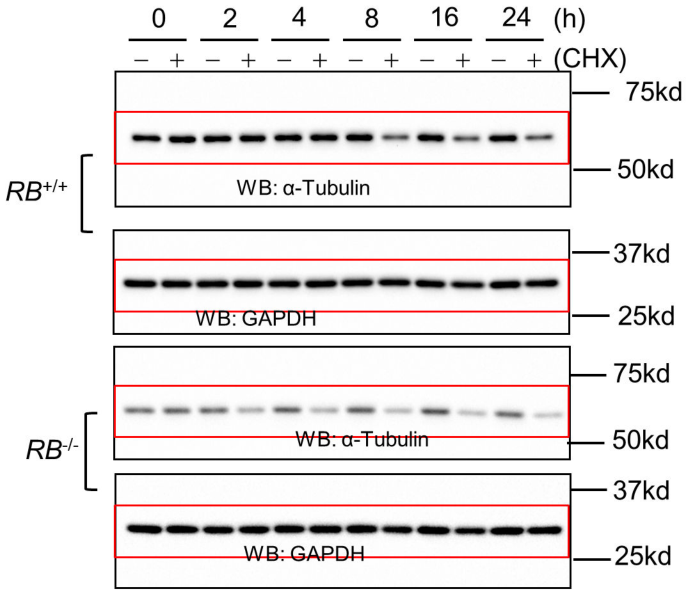

Figure 3g

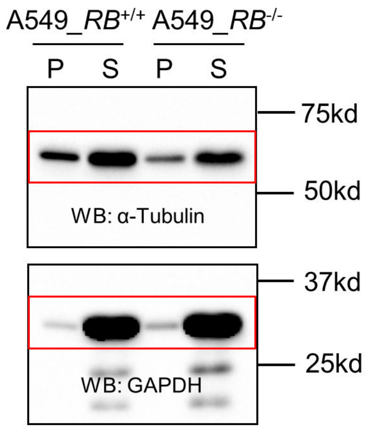

Figure 3h

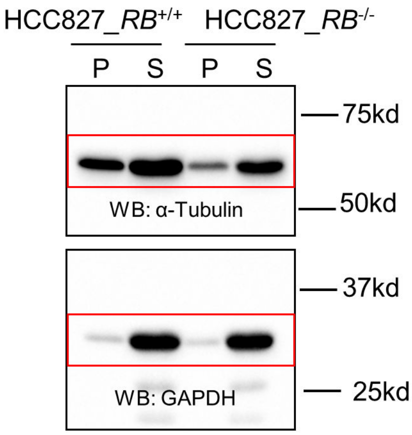

Supplementary Figure 15. Original Western blots shown in Figure 3. Each figure corresponds to the Western blots in the indicated Figure number.

Figure 3j

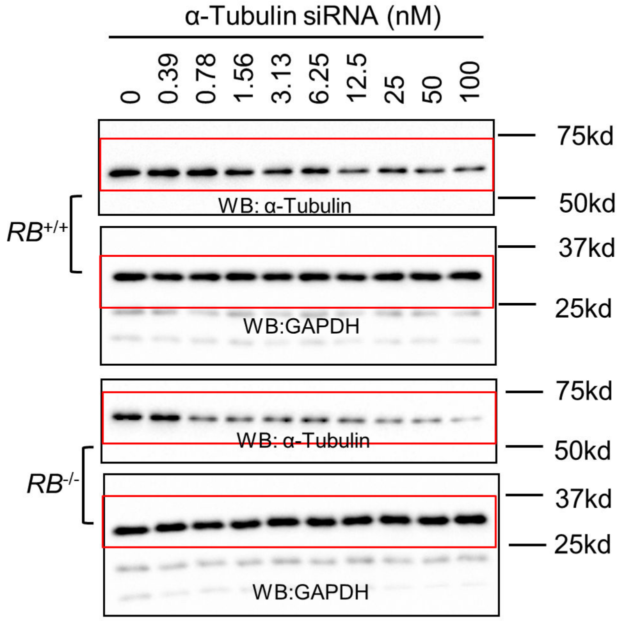

Figure 3l

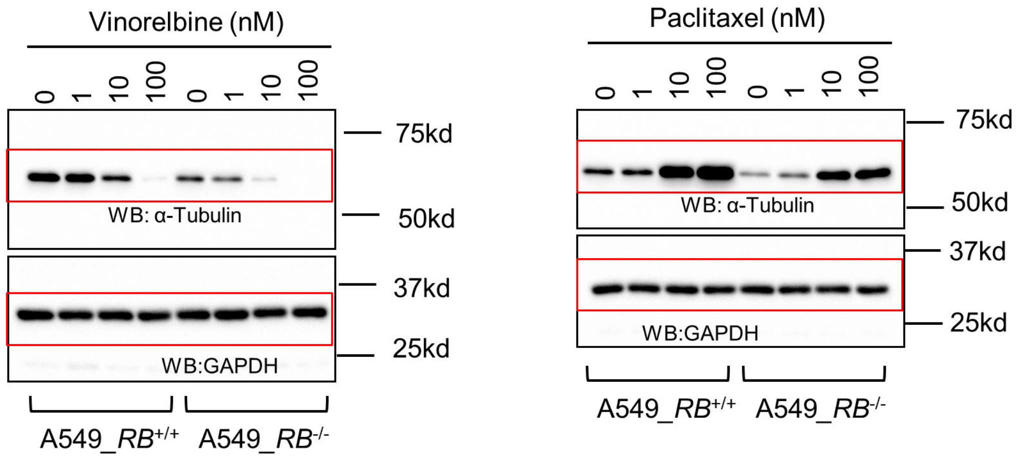

Supplementary Figure 16. Original Western blots shown in Figure 3. Each figure corresponds to the Western blots in the indicated Figure number.

Figure 4d

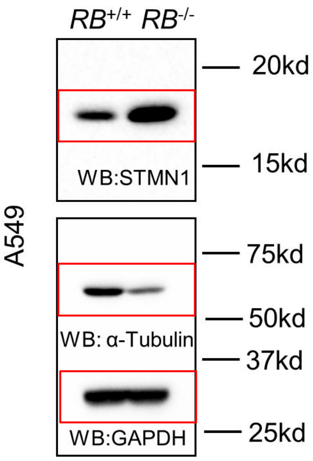

Figure 4e

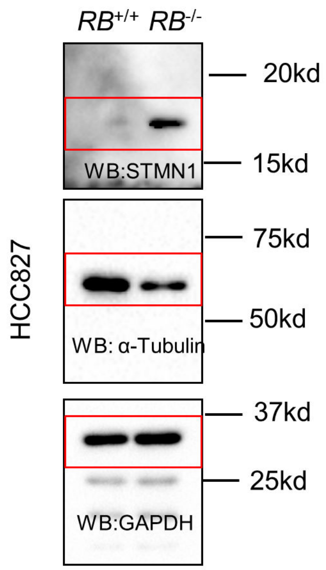

Figure 4f

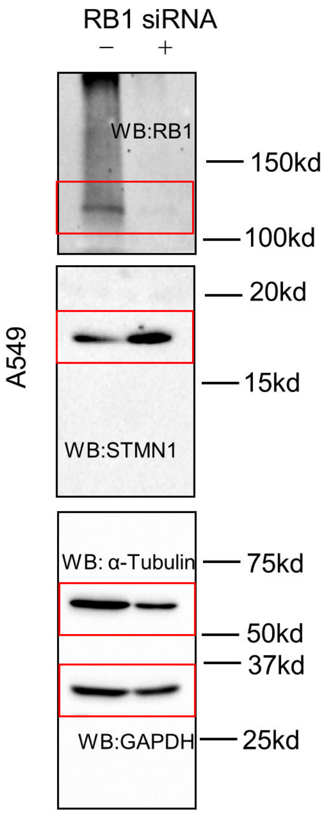

Figure 4g

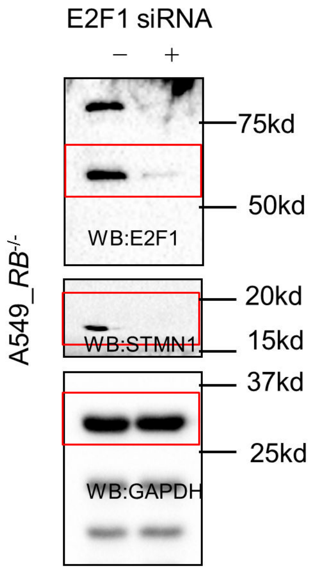

Figure 4k

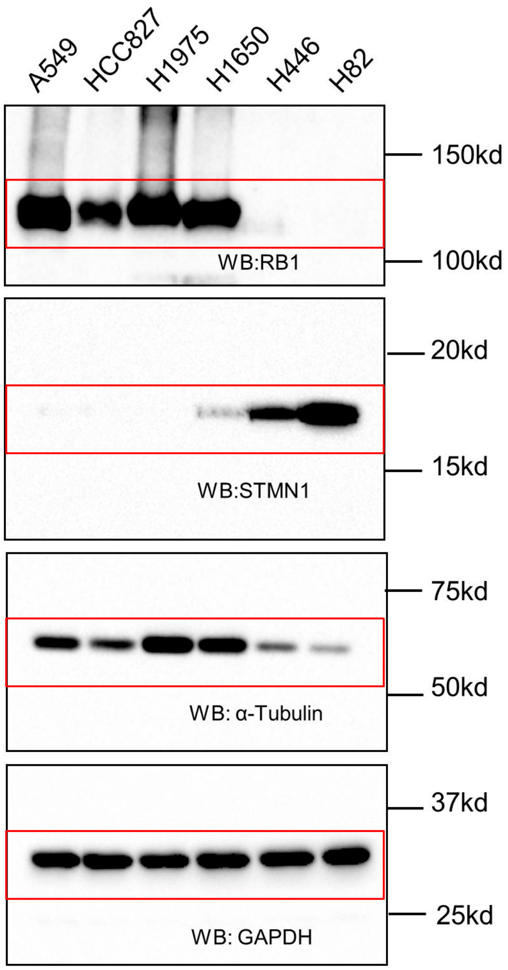

Supplementary Figure 17. Original Western blots shown in Figure 4. Each figure corresponds to the Western blots in the indicated Figure number.

Figure 5a

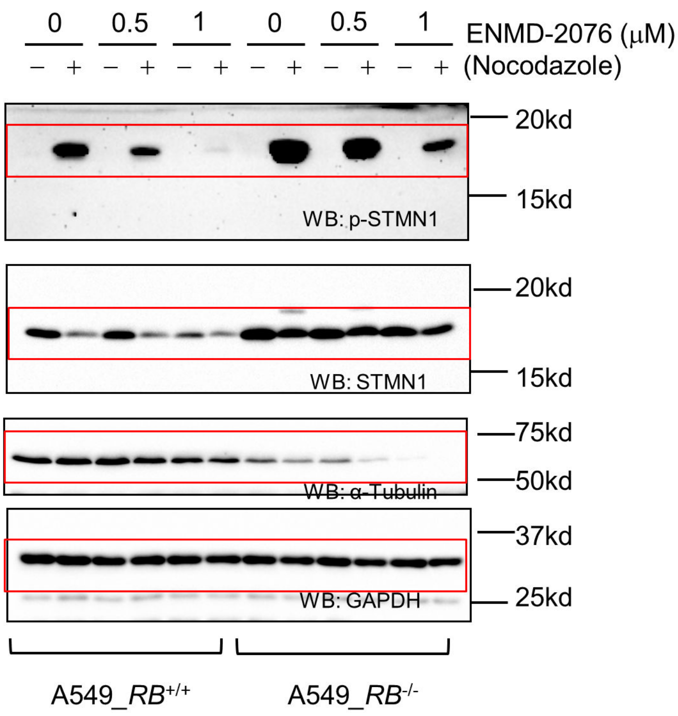

Figure 5b

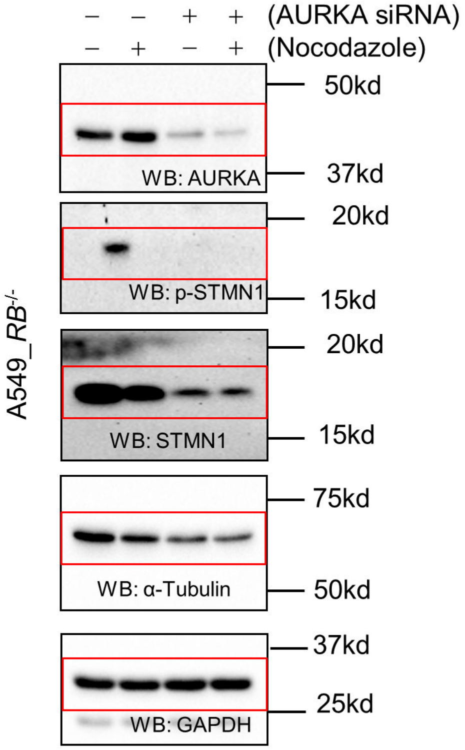

Figure 5c

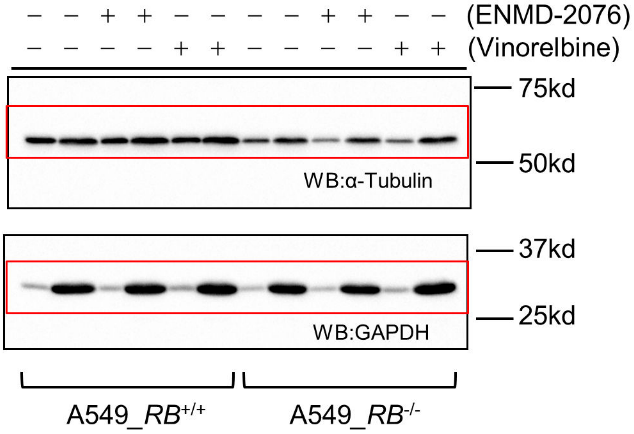

Figure 5d

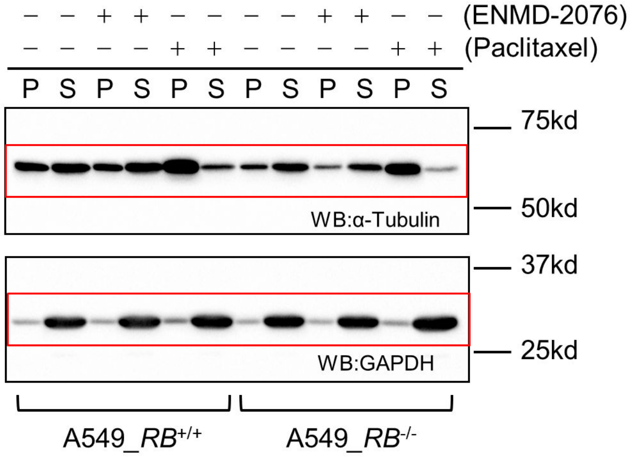

Supplementary Figure 18. Original Western blots shown in Figure 5. Each figure corresponds to the Western blots in the indicated Figure number.

Figure 5f

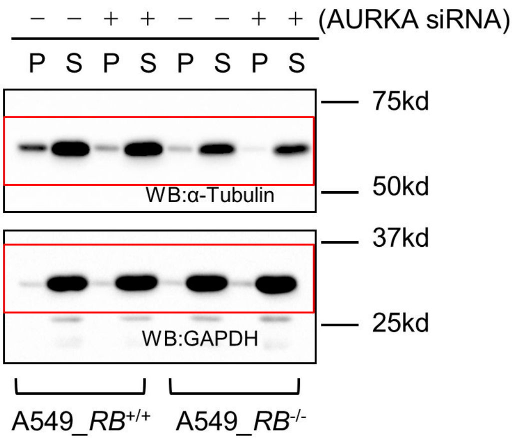

Figure 5h

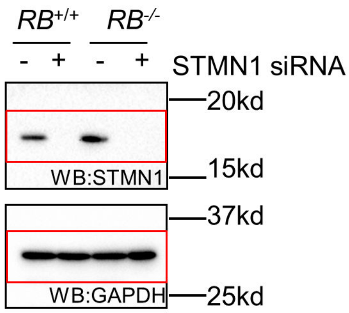

Figure 5k

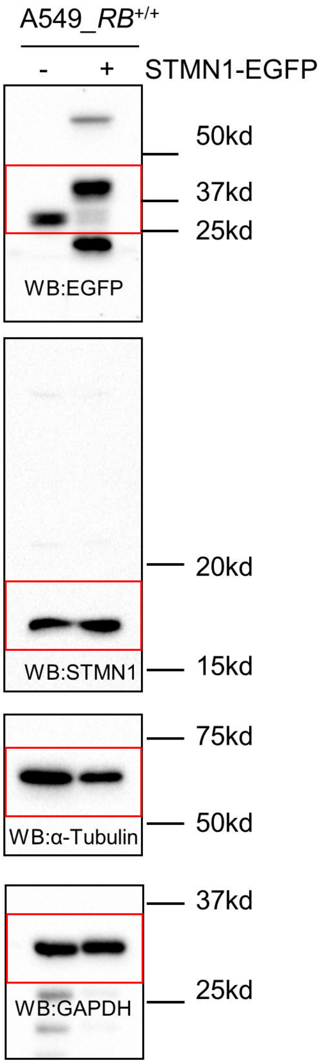

Figure 5i

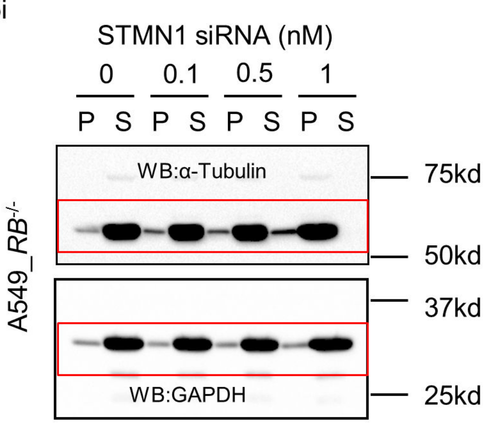

Figure 5l

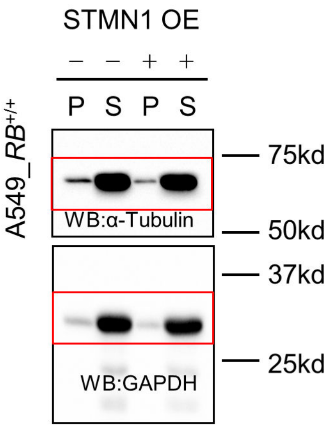

Supplementary Figure 19. Original Western blots shown in Figure 5. Each figure corresponds to the Western blots in the indicated Figure number.

Figure 7h

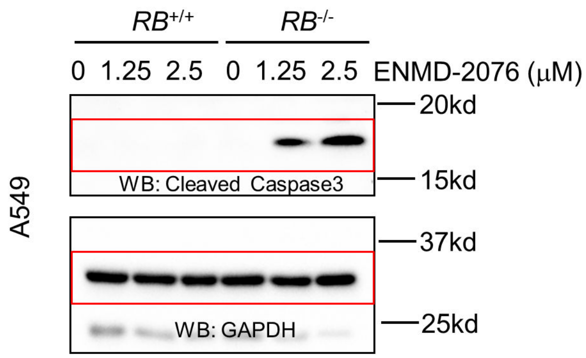

Figure 7i

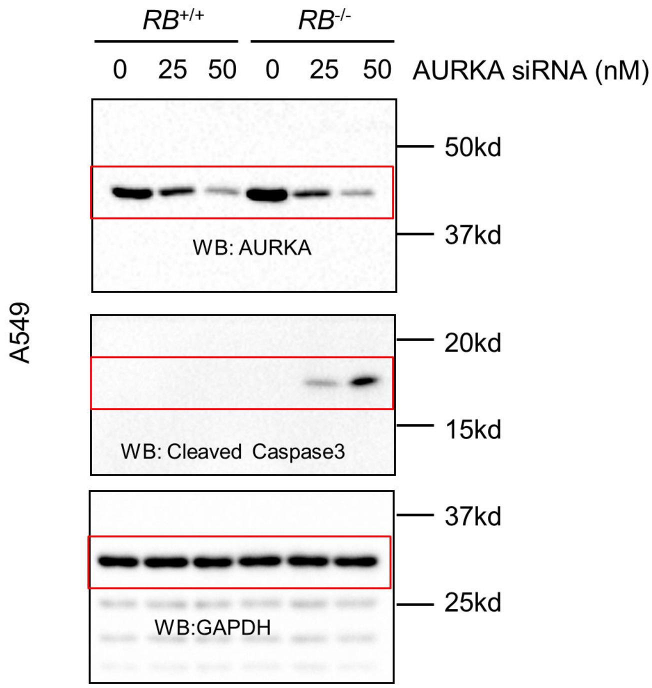

Figure 8e

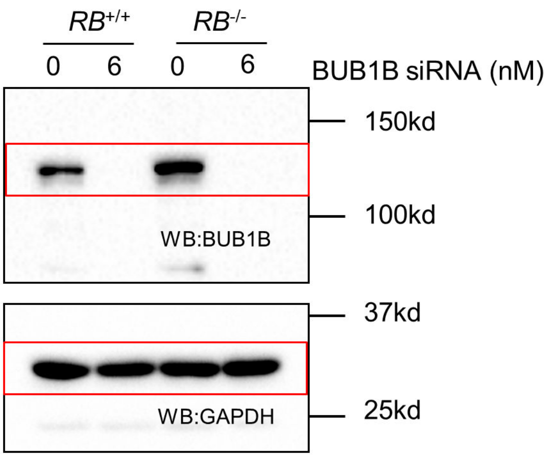

Supplementary Figure 20. Original Western blots shown in Figures 7-8. Each figure corresponds to the Western blots in the indicated Figure number.

Supplementary Figure 1e

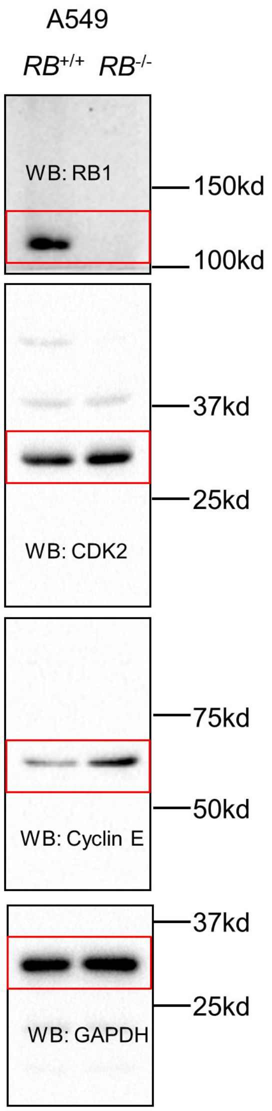

Supplementary Figure 2c

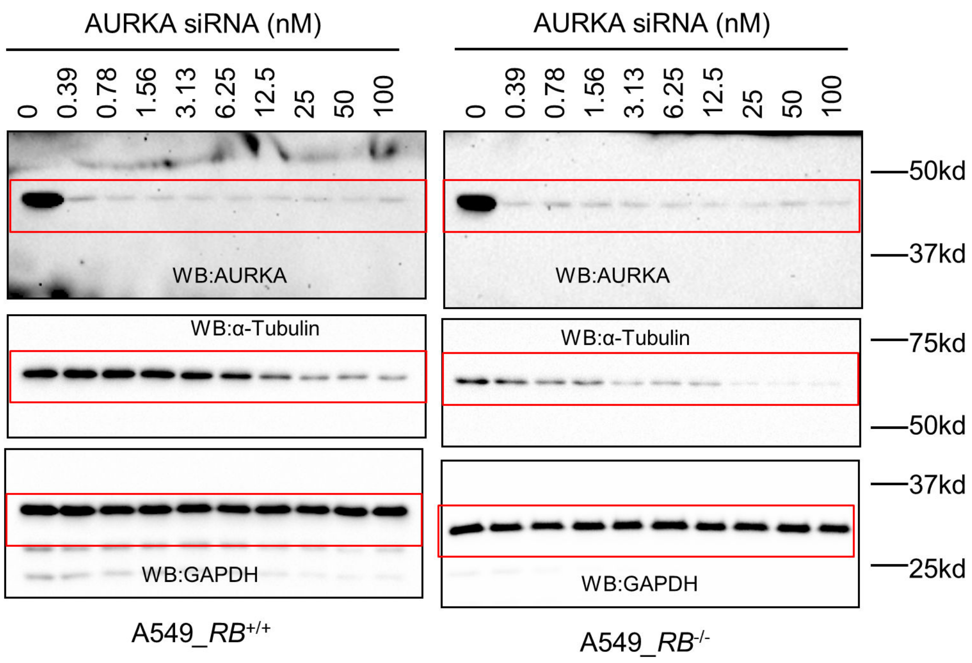

Supplementary Figure 5d

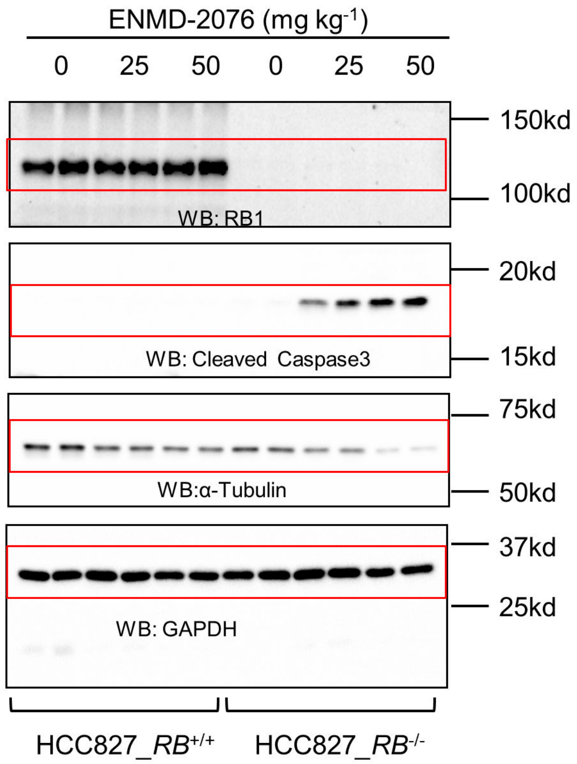

Supplementary Figure 21. Original Western blots shown in Supplementary Figures 1, 2 and 5. Each figure corresponds to the Western blots in the indicated Figure number.

Supplementary Figure 5e

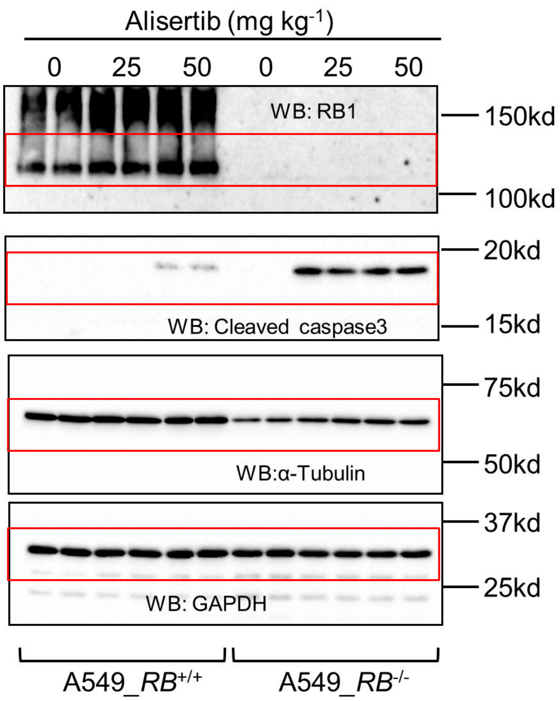

Supplementary Figure 5f

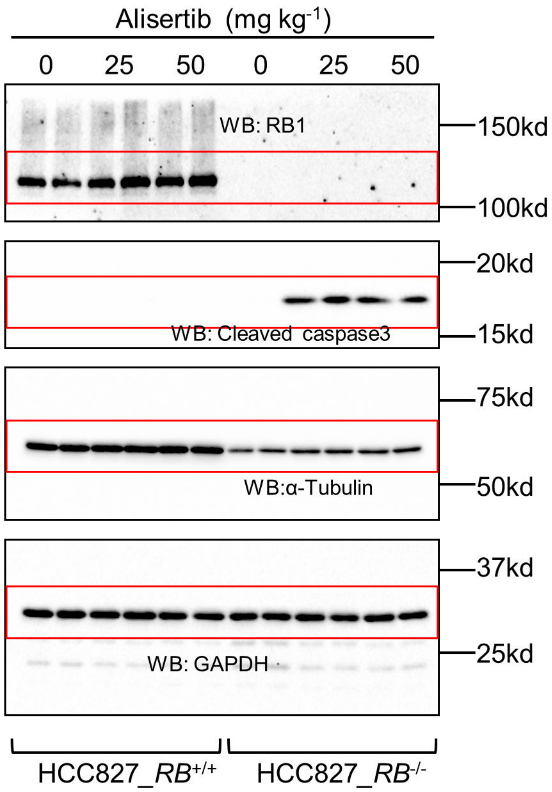

Supplementary Figure 5g

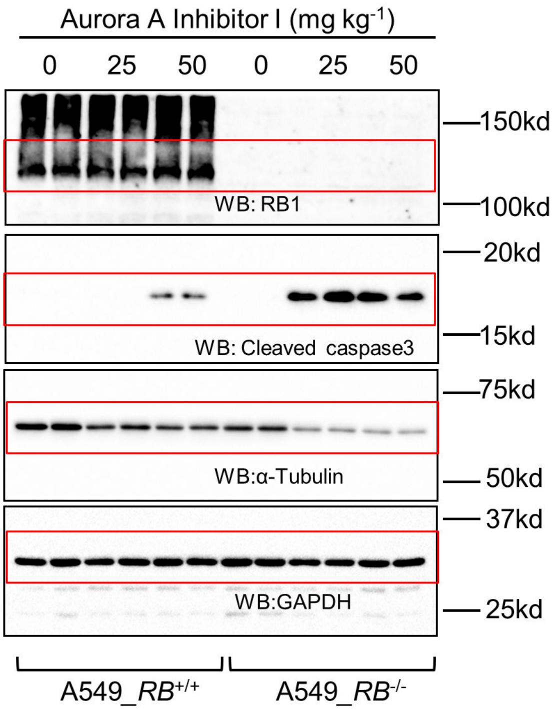

Supplementary Figure 5h

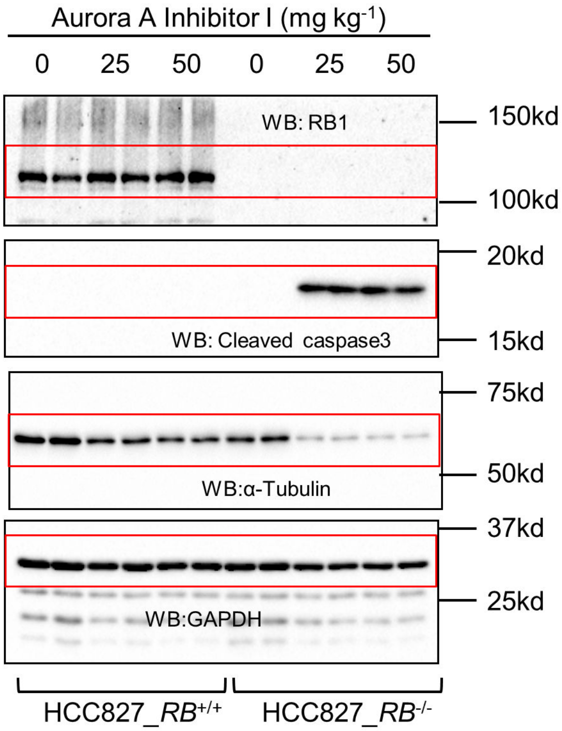

Supplementary Figure 22. Original Western blots shown in Supplementary Figure 5. Each figure corresponds to the Western blots in the indicated Figure number.

Supplementary Figure 7a

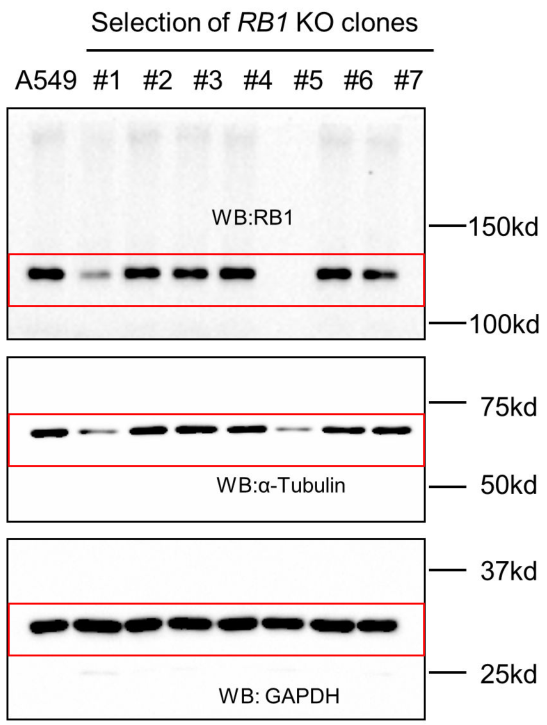

Supplementary Figure 7b

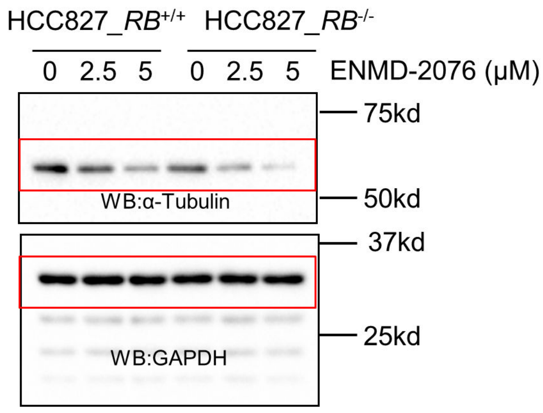

Supplementary Figure 7c

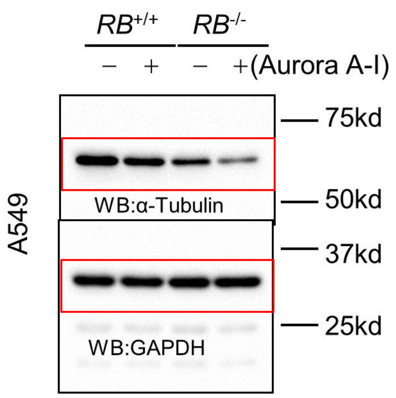

Supplementary Figure 7d

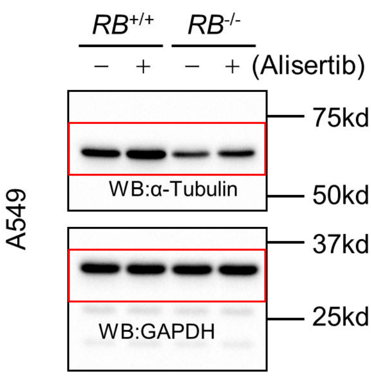

Supplementary Figure 23. Original Western blots shown in Supplementary Figure 7. Each figure corresponds to the Western blots in the indicated Figure number.

Supplementary Figure 8a

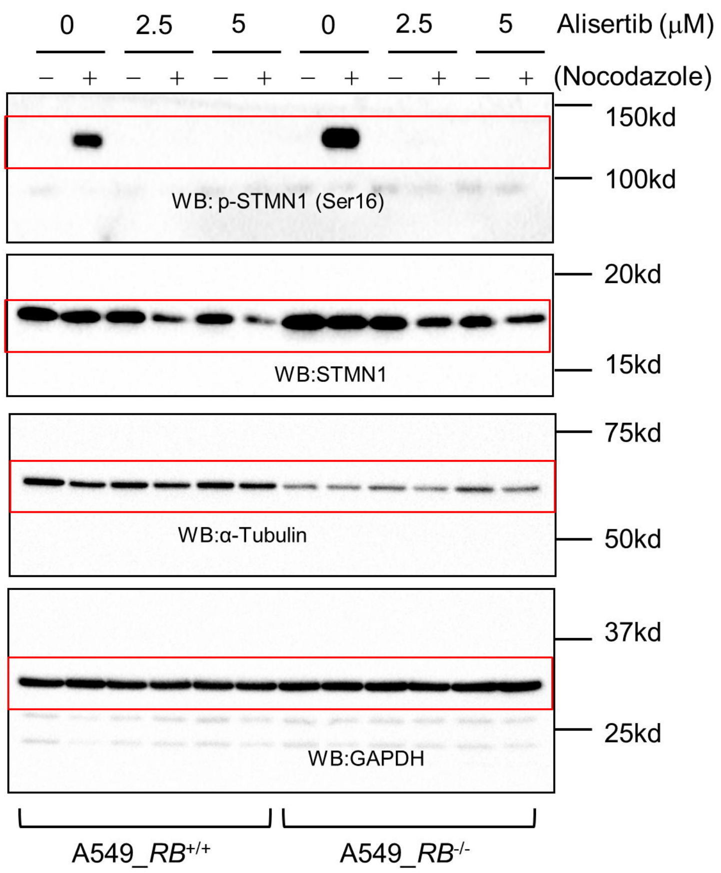

Supplementary Figure 8b

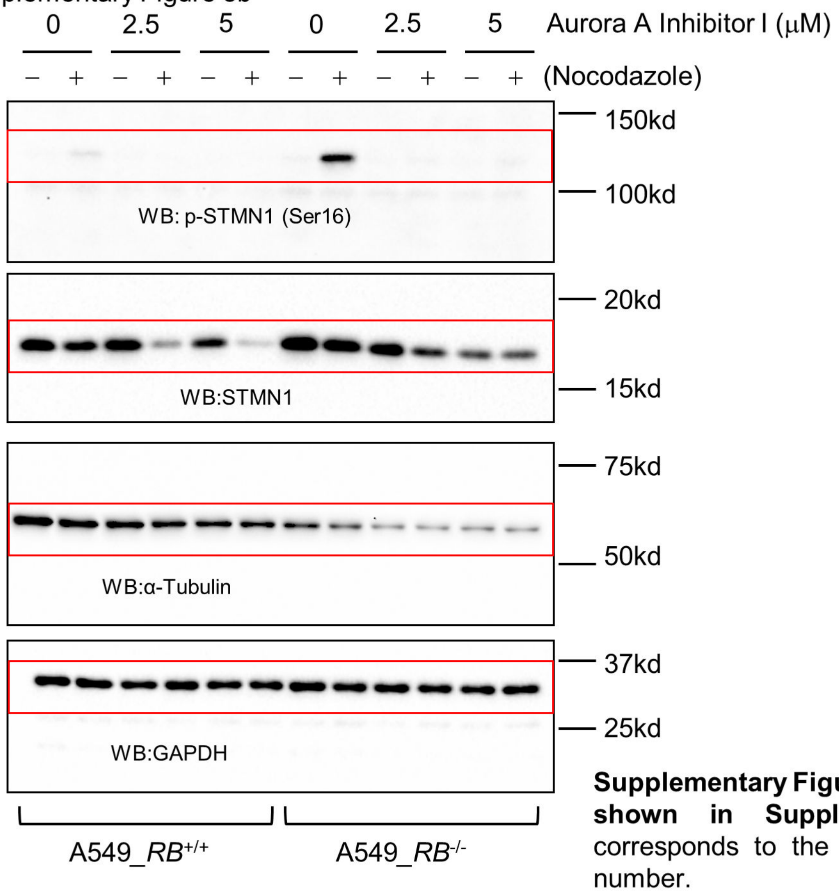

Supplementary Figure 24. Original Western blots shown in Supplementary Figure 8. Each figure corresponds to the Western blots in the indicated Figure number.

Supplementary Figure 9a

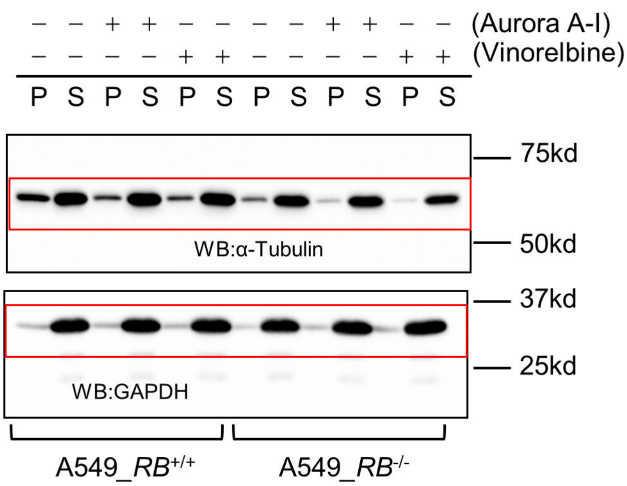

Supplementary Figure 9b

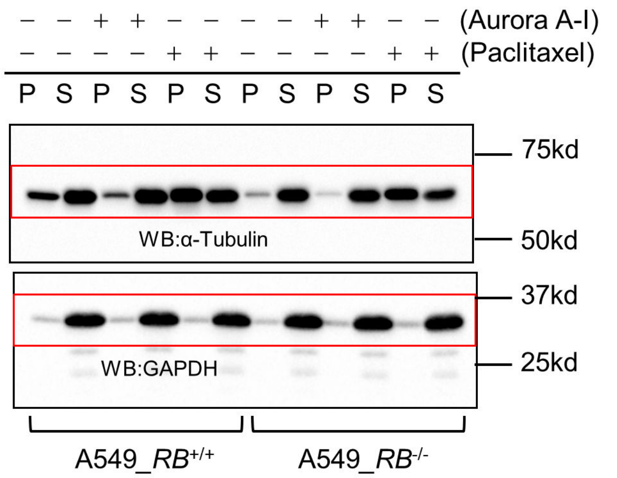

Supplementary Figure 9c

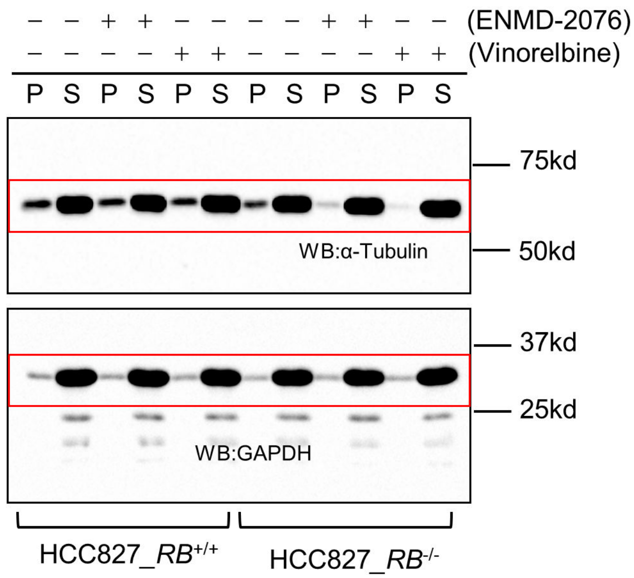

Supplementary Figure 9d

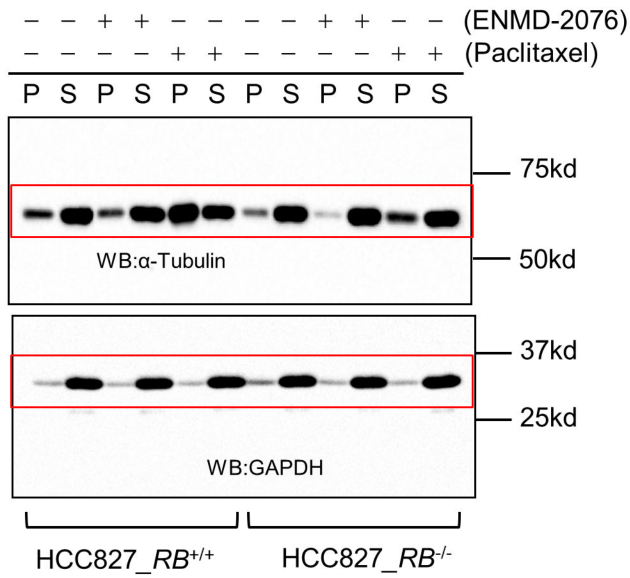

Supplementary Figure 9e

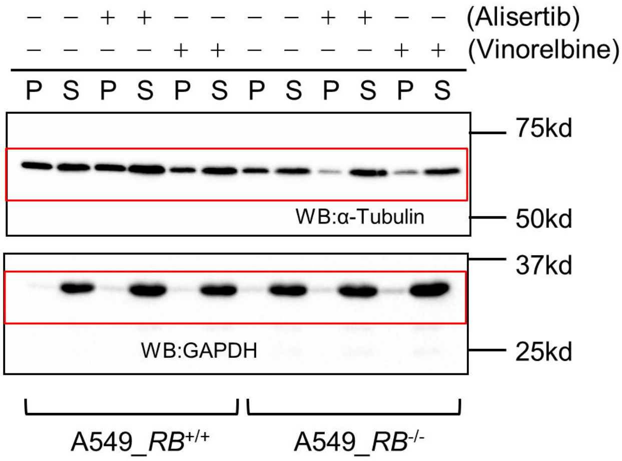

Supplementary Figure 9f

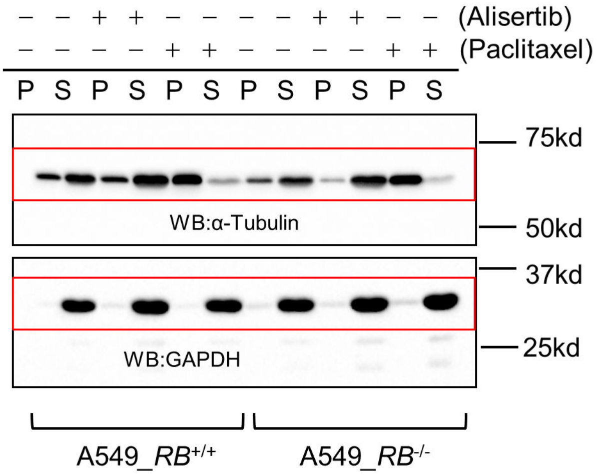

Supplementary Figure 25. Original Western blots shown in Supplementary Figure 9. Each figure corresponds to the Western blots in the indicated Figure number.

Supplementary Figure 10a

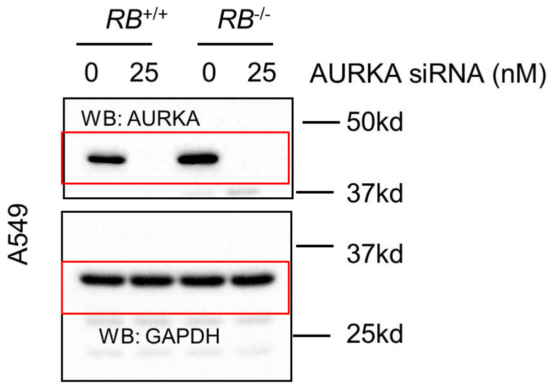

Supplementary Figure 12e

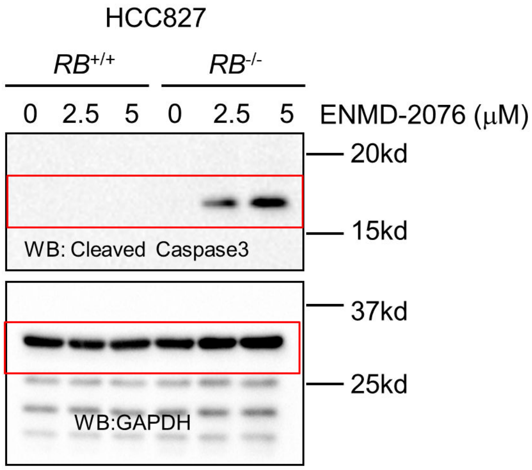

Supplementary Figure 12f

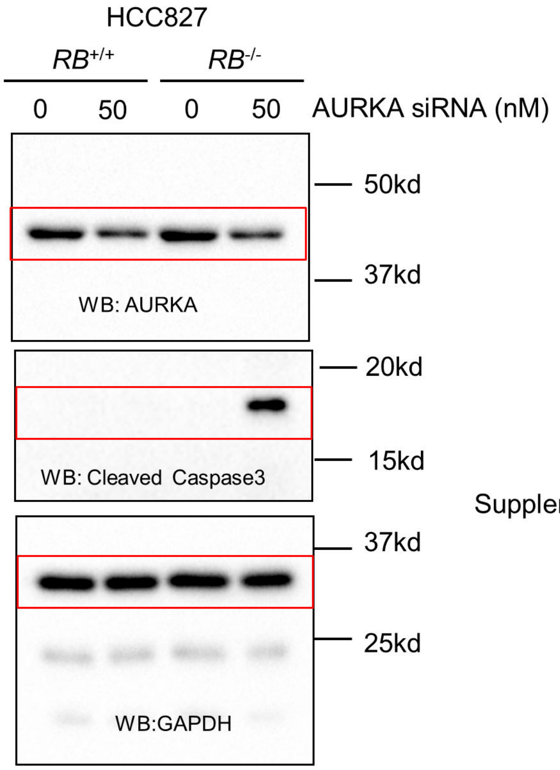

Supplementary Figure 13e

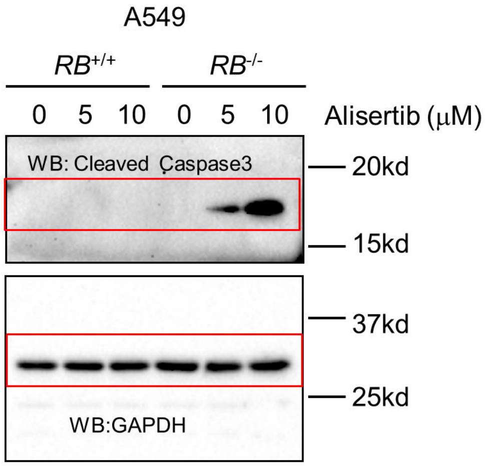

Supplementary Figure 13f

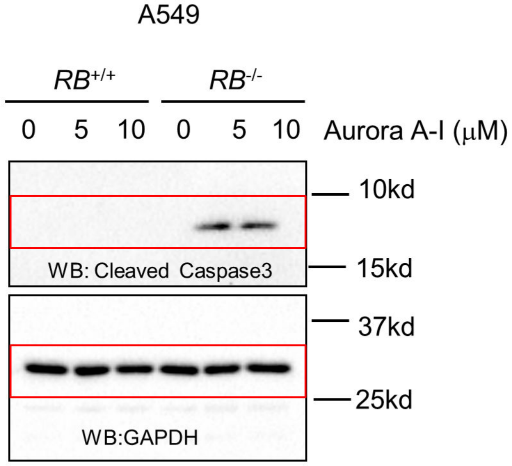

Supplementary Figure 26. Original Western blots shown in Supplementary Figures 10, 12 and 13. Each figure corresponds to the Western blots in the indicated Figure number.

Figure 7d

A549_*RB*$^{-/-}$
DMSO

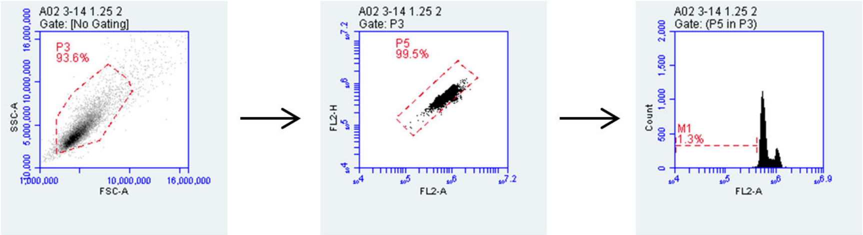

Figure 7f

A549_*RB*$^{-/-}$
DMSO

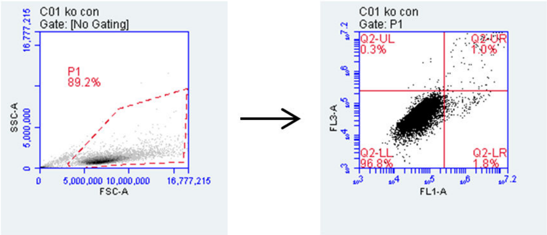

Supplementary Figure 12a

A549_*RB*$^{-/-}$
DMSO

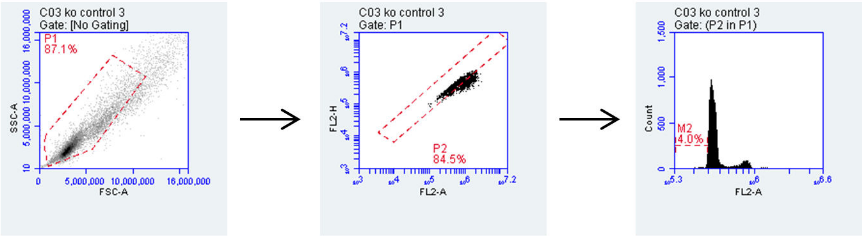

Supplementary Figure 12c

A549_*RB*$^{-/-}$
DMSO

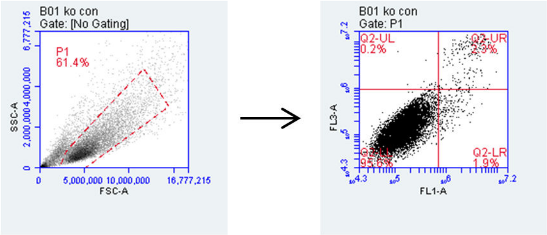

Supplementary Figure 27. FACS gating strategies used in Figure 7 and Supplementary Figure 12. Examples of FACS gating strategies for the cell cycle analysis (Figure 7d; Supplementary Figure 12a) and Annexin V-FITC apoptosis (Figure 7f; Supplementary Figure 12c) are shown.

Supplementary Figure 13a

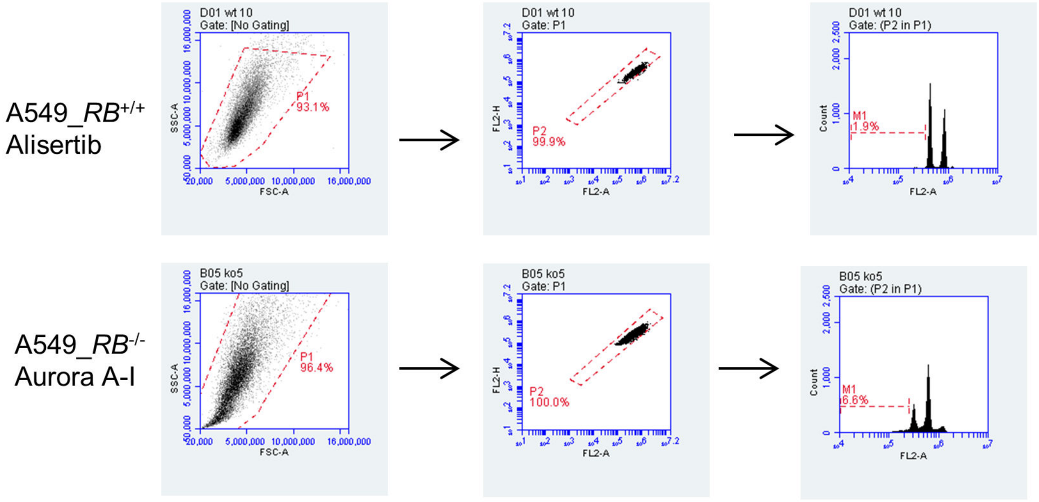

Supplementary Figure 13c

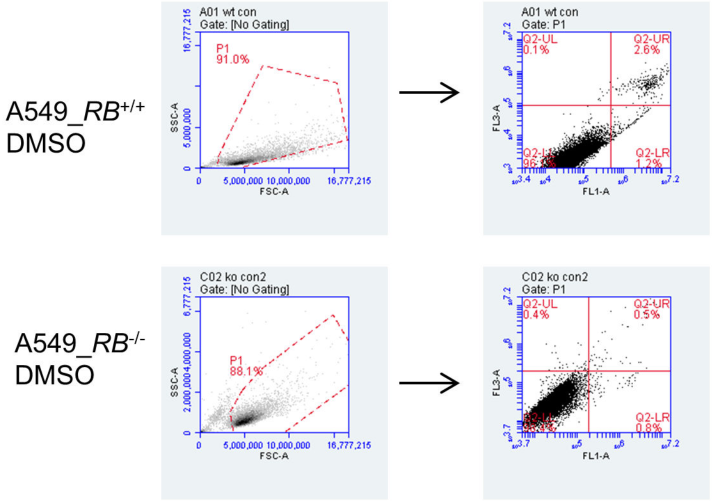

Supplementary Figure 28. FACS gating strategies used in Supplementary Figure 13. Examples of FACS gating strategies for the cell cycle analysis (Supplementary Figure 13a) and Annexin V-FITC apoptosis (Supplementary Figure 13c) are shown.

Supplementary Table 1. Antibodies used in this study.

Antibodies	Source	Identifier	Dilution factor
Rb (4H1) Mouse mAb	Cell Signaling Technology	9309S	1:1000
caspase-3 (31A1067)	Santa Cruz Biotechnology	sc-56053	1:100
Aurora A (D3E4Q) Rabbit mAb	Cell Signaling Technology	14475S	1:1000
Stathmin (D1Y5) Rabbit mAb	Cell Signaling Technology	13655S	1:1000
Phospho-Stathmin (Ser16) Antibody	Cell Signaling Technology	3353S	1:1000
E2F-1 Antibody	Cell Signaling Technology	3742S	1:1000
E2F-2 Antibody (TFE-25)	Santa Cruz Biotechnology	sc-9967	1:20
E2F3 Monoclonal Antibody (3E2F04 (PG37))	Thermo Fisher Scientific	MA5-11319	1:20
GFP Monoclonal Antibody	Immunoway Biotechnology Company	YM3124	1:1000
α Tubulin Antibody (B-7)	Santa Cruz Biotechnology	sc-5286	1:2000
GAPDH Antibody (G-9)	Santa Cruz Biotechnology	sc-365062	1:2500
Monoclonal Anti-Tubulin, Tyrosine antibody produced in mouse clone TUB-1A2	Sigma-Aldrich	T9028	1:100
BuB1B Monoclonal Antibody (OTI6E3), TrueMAB™	Thermo Fisher Scientific	TA500679	1:1000
Normal Mouse Ig G antibody produced in mouse	Sigma-Aldrich	M8695	1:25
Normal rabbit Ig G	Cell Signaling Technology	2729S	1:100
Purified Mouse Anti-Ki-67 Clone B56	BD Biosciences	550609	1:50
Goat anti-Mouse IgG (H+L) Secondary Antibody, HRP	Thermo Fisher Scientific	31430	1:1000
Goat anti-Rabbit IgG (H+L) Secondary Antibody, HRP	Thermo Fisher Scientific	31460	1:1000
Donkey anti-Mouse IgG (H+L) Highly Cross-Adsorbed Secondary Antibody, Alexa Fluor 488	Thermo Fisher Scientific	A21202	1:1000
Goat anti-Rabbit IgG (H+L) Cross-Adsorbed Secondary Antibody, Alexa Fluor 647	Thermo Fisher Scientific	A21244	1:1000

Supplementary Table 2. Oligonucleotides used in this study.

Oligo name	Sequence (5’ to 3’)	Notes
RB1 KO gRNA #1	5’-TGTTCGAGGTGAACCATTAA-3’	exon 8
RB1 KO gRNA #2	5’-CTCCTGTTCTGACCTCGCC-3’	exon 8
RB1 KO gRNA #3	5’-ATATGGTTCTTTGAGCAACA-3’	exon 7
exon-7 forward	5’-GGATATACTCTACCCTGCGATTT-3’	Genomic PCR
exon-7 reverse	5’-TCATCCTGTCAGCCTTAGAAC-3’	Genomic PCR
exon-8 forward	5’- AGCAGAGTAGAAGAGGGATGG-3’	Genomic PCR
exon-8 reverse	5’- GATTCCAGAGTGAGGGAGCTA-3’	Genomic PCR
HDR (loxP) reverse	5’- TGCCGACCCCTCCCCCCAAC-3’	Genomic PCR
TUBA1A forward	5’-TTGTAGACTTGGAACCCACAG-3’	qPCR
TUBA1A reverse	5'-ATCTCCTTGCCAATGGTGTAG-3'	qPCR
STMN1 forward	5'-AGCCCTCGGTCAAAAGAATC-3'	qPCR
STMN1 reverse	5'-TTCAAGACCTCAGCTTCATGG-3'	qPCR
STMN3 forward	5'-AGAAGCTCAACTACAAGATGGAG-3'	qPCR
STMN3 reverse	5'-CCTTAGCCCGACATCTCTTC-3'	qPCR
EML2 forward	5'-GCCATTCCAGTTTTATCACCC-3'	qPCR
EML2 reverse	5'-TCCATGTTCCTCACAGCATC-3'	qPCR
TPPP forward	5'-AAGAACTGGTCGAAGCTGTG-3'	qPCR
TPPP reverse	5'-ACTGCTCAAAGGTGATGGTC-3'	qPCR
TPPP3 forward	5'-AAGGTGGCTGACGGAAAG-3'	qPCR
TPPP3 reverse	5'-TTGCTCTTCCCCTTGAATCTC-3'	qPCR
NCALD forward	5'-TTCATCCGAGGAGCCAAAAG-3'	qPCR
NCALD reverse	5'-AATCAAAAGGGAACACAAGCAG-3'	qPCR
CLIP1 forward	5'-GTGGCGTGGAGTTAGATGAG-3'	qPCR
CLIP1 reverse	5'-TTTGGCTGGTGTAGTGGAAG-3'	qPCR
IFT81 forward	5'-CTGAGATTGACCCAAAGCAAC-3'	qPCR
IFT81 reverse	5'-CACCAAACCCTGACGAAAAG-3'	qPCR
PARK2 forward	5'-CGTGATTTGCTTAGACTGTTTCC-3'	qPCR
PARK2 reverse	5'-AGAATCCTGAAGTGATGGAGC-3'	qPCR
LRRK2 forward	5'-GATTGCCCCTGACTTGATTTTG-3'	qPCR
LRRK2 reverse	5'-CGGTAAACTGATCCAAAACTGC-3'	qPCR
GAPDH forward	5'-CCCTTCATTGACCTCAACTACA-3'	qPCR
GAPDH reverse	5'-ATGACAAGCTTCCCGTTCTC-3'	qPCR
STMN1 promoter F1	5‘-CCAAAGGCGATCAAATTCCAG-3’	qPCR
STMN1 promoter R1	5'-TTTCTAATCCACTCAGCCCAC-3'	qPCR
STMN1 promoter F2	5’-GCTCACAGGCTAAGTGAAAAC-3’	qPCR
STMN1 promoter R2	5’-AGCACACTACACAAAGCAAAC-3’	qPCR
STMN1 promoter F3	5'-CATGTGGCTCTACAAGGTGG-3'	qPCR
STMN1 promoter R3	5'-CGAGAACAAGGGCAGGG-3'	qPCR
Neutral region F	5’-GCCAACAGTCCAATAAAGCTG-3’	qPCR
Neutral region R	5’-TTGCCCATCTTATGTCCTGTG-3’	qPCR
