# Supplementary material for: Synthetic lethality of RB1 and aurora A is driven by stathmin-mediated disruption of microtubule dynamics
Source: Nat Commun. 2020 Oct 9;11:5105. doi: 10.1038/s41467-020-18872-0 (PMC7547687; doi:10.1038/s41467-020-18872-0)
Supplement: Supplementary file 3 — Reporting Summary [file 41467_2020_18872_MOESM3_ESM.pdf]

nature research

Corresponding author(s): Joong Sup Shim

Last updated by author(s): Aug 31, 2020

Reporting Summary

Nature Research wishes to improve the reproducibility of the work that we publish. This form provides structure for consistency and transparency in reporting. For further information on Nature Research policies, see Authors & Referees and the Editorial Policy Checklist.

Statistics

For all statistical analyses, confirm that the following items are present in the figure legend, table legend, main text, or Methods section.

n/a	Confirmed	
☐	☒	The exact sample size (*n*) for each experimental group/condition, given as a discrete number and unit of measurement
☐	☒	A statement on whether measurements were taken from distinct samples or whether the same sample was measured repeatedly
☐	☒	The statistical test(s) used AND whether they are one- or two-sided<br>*Only common tests should be described solely by name; describe more complex techniques in the Methods section.*
☒	☐	A description of all covariates tested
☒	☐	A description of any assumptions or corrections, such as tests of normality and adjustment for multiple comparisons
☐	☒	A full description of the statistical parameters including central tendency (e.g. means) or other basic estimates (e.g. regression coefficient) AND variation (e.g. standard deviation) or associated estimates of uncertainty (e.g. confidence intervals)
☐	☒	For null hypothesis testing, the test statistic (e.g. *F*, *t*, *r*) with confidence intervals, effect sizes, degrees of freedom and *P* value noted<br>*Give P values as exact values whenever suitable.*
☒	☐	For Bayesian analysis, information on the choice of priors and Markov chain Monte Carlo settings
☒	☐	For hierarchical and complex designs, identification of the appropriate level for tests and full reporting of outcomes
☐	☒	Estimates of effect sizes (e.g. Cohen's *d*, Pearson's *r*), indicating how they were calculated

Our web collection on statistics for biologists contains articles on many of the points above.

Software and code

Policy information about availability of computer code

Data collection	Image Lab (5.1 and 5.2.1) was used to acquire and analyze Western blot images. SoftMax Pro 6.3 was used to acquire the fluorescence of AlamarBlue® reagent. BD Accuri C6 Software (1.0.264.21) was used to acquire and analyze cell cycle and apoptosis data. 7500 software (v2.3) was used to acquire and analyze qPCR data. ZEN2012 (black version) was used to acquire and analyze confocal images.
Data analysis	ImageJ for Windows (bundled with 64-bit Java 1.8.0_112) was used to quantify images of Western blot bands and anti-tyr-tubulin immunostaining. GraphPad Prism 6.0 was used to calculate IC 50, plot the data and perform statistical analysis. BD Accuri C6TM Software (1.0.264.21) was used to quantify cell cycle and apoptosis. ZEN2012 (blue version) was used to process confocal images. FastQC (v0.11.5) was used for quality control for Raw RNA-Seq data. TopHat 2.1.1 was used to align and map the RNA-seq reads. Cufflinks 2.2.1 was used to define the gene expression. DAVID v6.8 (https://david.ncifcrf.gov) was used to perform gene ontology (GO) analysis. LinkFinder module was applied by LinkedOmics (http://www.linkedomics.org) to study the correlation between RB1 and stathmin protein expression in the TCGA-LUAD cohort.

For manuscripts utilizing custom algorithms or software that are central to the research but not yet described in published literature, software must be made available to editors/reviewers. We strongly encourage code deposition in a community repository (e.g. GitHub). See the Nature Research guidelines for submitting code & software for further information.

Data

Policy information about availability of data

All manuscripts must include a data availability statement. This statement should provide the following information, where applicable:

- Accession codes, unique identifiers, or web links for publicly available datasets
- A list of figures that have associated raw data
- A description of any restrictions on data availability

The RNA-Seq data have been deposited in the NCBI's Gene Expression Omnibus (GEO) database under the accession code GSE132722 (https://

www.ncbi.nlm.nih.gov/geo/query/acc.cgi?acc=GSE132722). The E2F1 ChIP-Seq data referenced during the study are available in a public repository from the ChIP-Atlas database (http://chip-atlas.org/). The proteomics data from 365 lung cancer patients (TCGA-LUAD cohort) are available in The Cancer Genome Atlas (TCGA) project (https://www.cancer.gov/about-nci/organization/ccg/research/structural-genomics/tcga) and are archived in LinkedOmics (http://www.linkedomics.org/) for expression analysis. All the other data supporting the findings of this study are available within the article and its supplementary information files and from the corresponding author upon reasonable request.

Field-specific reporting

Please select the one below that is the best fit for your research. If you are not sure, read the appropriate sections before making your selection.

☒ Life sciences ☐ Behavioural & social sciences ☐ Ecological, evolutionary & environmental sciences

For a reference copy of the document with all sections, see nature.com/documents/nr-reporting-summary-flat.pdf

Life sciences study design

All studies must disclose on these points even when the disclosure is negative.

Sample size	No statistical methods were used to predetermine sample size for animal experiments. The animal sample size (n=6) was chosen based on the previous publication (Wu and Yang, J Biopharm Stat, 2018, 28(3):437-450) describing the sample size calculation for tumor xenograft experiments, which were sufficient for statistical analysis. All in vitro experiments were performed in at least three biological replicates to allow for calculation of standard deviation, standard error of the mean and t-statistics for use in two-sided Student's t-test.
Data exclusions	No data were excluded from the analysis.
Replication	All the data with error bars were derived from experiments that were successfully repeated in at least three independent experiments.
Randomization	For all mice experiments, mice were randomly allocated prior to treatment. All other experiments were randomized using randomly assigned plates of cells per condition.
Blinding	Epigenetics drug screening, epigenetics siRNA screening and RNA-sequencing were blinded. All other works were not performed in a blinding manner, as each part of experiment was performed by one researcher and all biological samples were equally analyzed in each experiment.

Behavioural & social sciences study design

All studies must disclose on these points even when the disclosure is negative.

Study description	*Briefly describe the study type including whether data are quantitative, qualitative, or mixed-methods (e.g. qualitative cross-sectional, quantitative experimental, mixed-methods case study).*
Research sample	*State the research sample (e.g. Harvard university undergraduates, villagers in rural India) and provide relevant demographic information (e.g. age, sex) and indicate whether the sample is representative. Provide a rationale for the study sample chosen. For studies involving existing datasets, please describe the dataset and source.*
Sampling strategy	*Describe the sampling procedure (e.g. random, snowball, stratified, convenience). Describe the statistical methods that were used to predetermine sample size OR if no sample-size calculation was performed, describe how sample sizes were chosen and provide a rationale for why these sample sizes are sufficient. For qualitative data, please indicate whether data saturation was considered, and what criteria were used to decide that no further sampling was needed.*
Data collection	*Provide details about the data collection procedure, including the instruments or devices used to record the data (e.g. pen and paper, computer, eye tracker, video or audio equipment) whether anyone was present besides the participant(s) and the researcher, and whether the researcher was blind to experimental condition and/or the study hypothesis during data collection.*
Timing	*Indicate the start and stop dates of data collection. If there is a gap between collection periods, state the dates for each sample cohort.*
Data exclusions	*If no data were excluded from the analyses, state so OR if data were excluded, provide the exact number of exclusions and the rationale behind them, indicating whether exclusion criteria were pre-established.*
Non-participation	*State how many participants dropped out/declined participation and the reason(s) given OR provide response rate OR state that no participants dropped out/declined participation.*
Randomization	*If participants were not allocated into experimental groups, state so OR describe how participants were allocated to groups, and if allocation was not random, describe how covariates were controlled.*

Ecological, evolutionary & environmental sciences study design

All studies must disclose on these points even when the disclosure is negative.

Study description	*Briefly describe the study. For quantitative data include treatment factors and interactions, design structure (e.g. factorial, nested, hierarchical), nature and number of experimental units and replicates.*
Research sample	*Describe the research sample (e.g. a group of tagged Passer domesticus, all Stenocereus thurberi within Organ Pipe Cactus National Monument), and provide a rationale for the sample choice. When relevant, describe the organism taxa, source, sex, age range and any manipulations. State what population the sample is meant to represent when applicable. For studies involving existing datasets, describe the data and its source.*
Sampling strategy	*Note the sampling procedure. Describe the statistical methods that were used to predetermine sample size OR if no sample-size calculation was performed, describe how sample sizes were chosen and provide a rationale for why these sample sizes are sufficient.*
Data collection	*Describe the data collection procedure, including who recorded the data and how.*
Timing and spatial scale	*Indicate the start and stop dates of data collection, noting the frequency and periodicity of sampling and providing a rationale for these choices. If there is a gap between collection periods, state the dates for each sample cohort. Specify the spatial scale from which the data are taken*
Data exclusions	*If no data were excluded from the analyses, state so OR if data were excluded, describe the exclusions and the rationale behind them, indicating whether exclusion criteria were pre-established.*
Reproducibility	*Describe the measures taken to verify the reproducibility of experimental findings. For each experiment, note whether any attempts to repeat the experiment failed OR state that all attempts to repeat the experiment were successful.*
Randomization	*Describe how samples/organisms/participants were allocated into groups. If allocation was not random, describe how covariates were controlled. If this is not relevant to your study, explain why.*
Blinding	*Describe the extent of blinding used during data acquisition and analysis. If blinding was not possible, describe why OR explain why blinding was not relevant to your study.*

Did the study involve field work? ☐ Yes ☒ No

Field work, collection and transport

Field conditions	*Describe the study conditions for field work, providing relevant parameters (e.g. temperature, rainfall).*
Location	*State the location of the sampling or experiment, providing relevant parameters (e.g. latitude and longitude, elevation, water depth).*
Access and import/export	*Describe the efforts you have made to access habitats and to collect and import/export your samples in a responsible manner and in compliance with local, national and international laws, noting any permits that were obtained (give the name of the issuing authority, the date of issue, and any identifying information).*
Disturbance	*Describe any disturbance caused by the study and how it was minimized.*

Reporting for specific materials, systems and methods

We require information from authors about some types of materials, experimental systems and methods used in many studies. Here, indicate whether each material, system or method listed is relevant to your study. If you are not sure if a list item applies to your research, read the appropriate section before selecting a response.

Materials & experimental systems

n/a	Involved in the study	
☐	☒	Antibodies
☐	☒	Eukaryotic cell lines
☒	☐	Palaeontology
☐	☒	Animals and other organisms
☒	☐	Human research participants
☒	☐	Clinical data

Methods

n/a	Involved in the study	
☒	☐	ChIP-seq
☐	☒	Flow cytometry
☒	☐	MRI-based neuroimaging

Antibodies

Antibodies used

All the antibody information (total 20 antibodies) and dilution factors are provided in 'Methods' section and in Supplementary Table 1.

Validation

RB (4H1) Mouse mAb, CST, #9309: WB, IP, IHC-P, IF-IC, F, ChIP. H Mk B Pg. 312 citations; CST website antibody validation: 1) Western blot analysis of extracts from HeLa cells or Rb knock-out cells shows single band at expected MW of 110 kDa. The absence of signal in the Rb knock-out HeLa cells confirms specificity of the antibody for Rb. 2) Western blot analysis of extracts from HeLa cells, transfected with Control siRNA, Rb siRNA. The Rb (4H1) Mouse mAb confirms silencing of Rb expression. 3) Western blot analysis of extracts from COS-7 cells, untreated or hydroxyurea-treated (G1/S). Hydroxyurea-treated (G1/S) shows expected increase of Rb band. 4) Immunohistochemical analysis of paraffin-embedded human breast carcinoma shows nuclear localization. 5) Immunohistochemical analysis of paraffin-embedded human lung carcinoma shows nuclear localization of Rb. 6) Confocal immunofluorescent image of SH-SY5Y cells shows nuclear localization of Rb.

Caspase-3 (31A1067), SC, sc-56053, WB, IP, IF, IHC, ELISA. 91 citations; SC website antibody validation: 1) Western blot analysis of caspase-3 expression in CCRF-CEM, HuT 78, CCRF-HSB-2, PC-3, HeLa and U-698-M whole cell lysates shows at expected MW of 32 kDa. 2) Western blot analysis of caspase-3 expression in untreated and Staurosporine treated HeLa whole cell lysates shows cleaved caspase-3 expression in Staurosporine treated HeLa at expected MW of 17 kDa.

Aurora A (D3E4Q) Rabbit mAb, CST, #14475: WB, IP, IF-IC, F. 22 citations; CST website antibody validation: 1) Western blot analysis of extracts from HT-29 cells, untreated or synchronized in mitosis by treatment with thymidine and Nocodazole shows single band at expected MW of 48 kDa. 2) Immunoprecipitation of Aurora A from HeLa cell extracts. Western blot analysis shows the band at expected MW of 48 kDa. 3) Confocal immunofluorescent analysis of HeLa cells shows the centromere localization of using Aurora A. 4) Flow cytometric analysis of Jurkat cells using Aurora A antibody and Propidium Iodide (PI)/RNase Staining Solution shows Aurora A positive cells.

Stathmin (D1Y5) Rabbit mAb, CST, #1365: WB, IHC-P, IF-IC. 3 citations; CST website antibody validation: 1) Western blot analysis of extracts from various cell lines shows single band at expected MW of 17 kDa. 2) Immunohistochemical analysis of paraffin-embedded human breast carcinoma shows cytoplasmic staining of stathmin. 3) Immunohistochemical analysis of paraffin-embedded mouse kidney shows cytoplasmic staining of stathmin. 4) Immunohistochemical analysis of paraffin-embedded human lung carcinoma shows cytoplasmic staining of stathmin. 5) Immunohistochemical analysis of paraffin-embedded human lymph node shows cytoplasmic staining of stathmin. 6) Confocal immunofluorescent analysis of C2C12 cells shows cytoplasmic staining of stathmin.

Phospho-Stathmin (Ser16) Antibody, CST, #3353: WB. 5 citations; CST website antibody validation: Western blot analysis of extracts from HeLa and C2C12 cells, untreated or nocodazole-treated, shows expected band at MW of 19 and 20 kDa.

E2F1 Antibody, CST, #3742: WB, ChIP. 114 citations; CST website antibody validation: 1) Western Blot analysis of extracts from HeLa, SK-N-MC, and A-673 cells shows the band at expected MW of 70 kDa. 2) Western blot analysis of extracts from HeLa cells, untransfected or transfected with E2F1 siRNA to silence E2F1 protein expression, shows expected reduction of E2F1 bands. 3) Chromatin immunoprecipitations were performed with cross-linked chromatin from Raji cells and either E2F-1 Antibody or Normal Rabbit IgG. The enriched DNA was quantified by real-time PCR using SimpleChIP® Human Timeless Intron 1 Primers and human DHFR promoter primers. Real-time PCR shows that DNA enrichment of E2F1 signal versus the negative control IgG.

E2F2 Antibody (TFE-25), SC, sc-9967: CHIP, GS, WB, IHC (P), ELISA B, IF and FCM. 28 citations; SC website antibody validation: 1) Direct western blot analysis of E2F-2 expression in KNRK nuclear extract and PC-12 and Ramos whole cell lysates shows the band at expected MW of 55 kDa. 2) Immunoperoxidase staining of formalin fixed, paraffin-embedded human placenta tissue shows nuclear and cytoplasmic staining of trophoblastic cells at low and high magnification.

E2F3 Monoclonal Antibody (3E2F04 (PG37)), Thermo Fisher Scientific, MA5-11319: CHIP, GS, IF, WB. 3 citations; Thermo Fisher Scientific website antibody validation: 1) Western blot analysis of whole cell extracts of HeLa, SH-SY5Y, U-87MG, MDA-MB-231, PC-3 shows the bands at expected MW of 37 and 49 kDa. 2) Chromatin Immunoprecipitation (ChIP) was performed using Anti-E2F3 Transcription Factor Mouse Monoclonal Antibody and Normal Mouse IgG. The enriched DNA was quantified by real-time PCR using the primer pairs for the promoter of active E2F3, ATATD2, RAD51 gene, used as positive control target, and the SAT2, used as negative control target. Real-time PCR shows that DNA enrichment of E2F3 signal versus the negative control IgG.

GFP Monoclonal Antibody, Immunoway Biotechnology Company, YM3124: WB, IF/ICC, IP. Immunoway Biotechnology Company website antibody validation: 1) Western blot analysis of GFP transfected Hela shows the bands at expected MW of 26 kDa. 2) Western blot analysis of Input and IP products shows the bands at expected MW of 26 kDa.

α-Tubulin Antibody (B-7), SC, sc-5286: WB, IP, IHC (P), IF, FCM. 1203 citations; SC website antibody validation: 1) Western blot analysis of α Tubulin expression in K-562, HEL 92.1.7, RAW 264.7, C2C12, PC-12 and A-10 whole cell lysates shows the bands at expected MW of 55 kDa. 2) Immunofluorescence staining of formalin-fixed HeLa cells shows cytoplasmic staining of α Tubulin. 3) Immunoperoxidase staining of formalin fixed, paraffinembedded human heart muscle tissue shows cytoplasmic staining of myocytes.

GAPDH Antibody (G-9), SC, sc-365062: WB, IP, IF, IHC (P), ELISA. 927 citations; SC website antibody validation: 1) Simultaneous direct near-infrared western blot analysis shows the bands at expected MW of 36 kDa. 2) Direct immunofluorescence staining of formalin-fixed SW480 cells shows membrane, cytoplasmic and nuclear localization of GAPDH. 3) Direct immunoperoxidase staining of formalin fixed, paraffin-embedded human epididymis tissue shows cytoplasmic and nuclear staining of glandular cells.

Monoclonal Anti-Tubulin, Tyrosine antibody produced in mouse clone TUB-1A2, Sigma-Aldrich, T9028: IF, WB, microarray. 117 citations; Sigma-Aldrich website antibody validation: 1) Western blot analysis of Tyrosine-Tubulin shows the bands at expected

MW of 55 kDa. 2) Immunohistochemistry analysis of Sertoli cell microtubules shows cytoplasmic localization of Tyrosine-Tubulin. 3) Immunofluorescence analysis of Chicken fibroblasts cells shows cytoplasmic localization of Tyrosine-Tubulin.

BuB1B Monoclonal Antibody (OTI6E3), TrueMAB™, Thermo Fisher Scientific, TA500679: FC, IF, WB, ICC. Thermo Fisher Scientific website antibody validation: 1) Immunofluorescent staining of COS7 cells transiently transfected by pCMV6-ENTRY BUB1B shows cytoplasmic localization of BUB1B. 2) Western blot analysis of extracts from 9 different cell lines shows the bands at expected MW of 119.3 kDa. 3) Western blot analysis of HEK293T cells transfected with the pCMV6-ENTRY control or pCMV6-ENTRY BUB1B shows the bands at expected MW of 119.3 kDa. 4) Flow cytometric Analysis of Jurkat cells using anti-BUB1B antibody shows peak shift compared to a nonspecific negative control antibody. 5) Flow cytometric Analysis of Hela cells using anti-BUB1B antibody shows peak shift compared to a nonspecific negative control antibody. 6) HEK293T cells transfected with overexpress plasmid shows peak shift compared to empty vector control plasmid.

Purified Mouse Anti-Ki-67 Clone B56, BD Biosciences, #550609: IHC.14 citations; BD Biosciences website antibody validation: Immunohistochemistry of Ki-67 positive cells. Formalin-fixed paraffin embedded sections of normal human tonsil were reacted with the anti-Ki-67 antibody. Proliferating cells expressing Ki-67 can be identifed by the intense brown labeling of their cell nuclei.

Eukaryotic cell lines

Policy information about cell lines

Cell line source(s)	The human lung cancer cell lines, A549, HCC827, NCI-H1650, NCI-H1975, NCI-H446 and NCI-H82 and the human breast cancer cell line, MDA-MB-468 were obtained from ATCC.
Authentication	Yes, by morphology, karyotyping and STR profiling provided by ATCC.
Mycoplasma contamination	All the cells tested are negative for mycoplasma contamination. The information is described in 'Methods' section.
Commonly misidentified lines (See ICLAC register)	None of cell lines used in this study are listed in the database of commonly misidentified cell lines maintained by ICLAC.

Palaeontology

Specimen provenance	*Provide provenance information for specimens and describe permits that were obtained for the work (including the name of the issuing authority, the date of issue, and any identifying information).*
Specimen deposition	*Indicate where the specimens have been deposited to permit free access by other researchers.*
Dating methods	*If new dates are provided, describe how they were obtained (e.g. collection, storage, sample pretreatment and measurement), where they were obtained (i.e. lab name), the calibration program and the protocol for quality assurance OR state that no new dates are provided.*

☐ Tick this box to confirm that the raw and calibrated dates are available in the paper or in Supplementary Information.

Animals and other organisms

Policy information about studies involving animals; ARRIVE guidelines recommended for reporting animal research

Laboratory animals	All animals were maintained in the University of Macau, specific pathogen free (SPF) Animal Facility. Six- to eight-week female Athymic Nude mice (The Jackson Laboratory) were used for tumor xenografts. Mice were housed in a fully climate-controlled room at constant temperature and humidity on a 12:12 h light/dark cycle with free access to food and water.
Wild animals	This study did not involve wild animals.
Field-collected samples	This study did not involve samples collected from the field.
Ethics oversight	All animal procedures were approved by the Animal Research Ethics Committee of the University of Macau. The information is provided in 'Methods' section.

Note that full information on the approval of the study protocol must also be provided in the manuscript.

Human research participants

Policy information about studies involving human research participants

Population characteristics	*Describe the covariate-relevant population characteristics of the human research participants (e.g. age, gender, genotypic information, past and current diagnosis and treatment categories). If you filled out the behavioural & social sciences study design questions and have nothing to add here, write "See above."*
Recruitment	*Describe how participants were recruited. Outline any potential self-selection bias or other biases that may be present and how these are likely to impact results.*

Ethics oversight | *Identify the organization(s) that approved the study protocol.*

Note that full information on the approval of the study protocol must also be provided in the manuscript.

Clinical data

Policy information about clinical studies

All manuscripts should comply with the ICMJE guidelines for publication of clinical research and a completed CONSORT checklist must be included with all submissions.

Clinical trial registration | *Provide the trial registration number from ClinicalTrials.gov or an equivalent agency.*

Study protocol | *Note where the full trial protocol can be accessed OR if not available, explain why.*

Data collection | *Describe the settings and locales of data collection, noting the time periods of recruitment and data collection.*

Outcomes | *Describe how you pre-defined primary and secondary outcome measures and how you assessed these measures.*

ChIP-seq

Data deposition

☐ Confirm that both raw and final processed data have been deposited in a public database such as GEO.

☐ Confirm that you have deposited or provided access to graph files (e.g. BED files) for the called peaks.

Data access links
May remain private before publication. | *For "Initial submission" or "Revised version" documents, provide reviewer access links. For your "Final submission" document, provide a link to the deposited data.*

Files in database submission | *Provide a list of all files available in the database submission.*

Genome browser session
(e.g. UCSC) | *Provide a link to an anonymized genome browser session for "Initial submission" and "Revised version" documents only, to enable peer review. Write "no longer applicable" for "Final submission" documents.*

Methodology

Replicates | *Describe the experimental replicates, specifying number, type and replicate agreement.*

Sequencing depth | *Describe the sequencing depth for each experiment, providing the total number of reads, uniquely mapped reads, length of reads and whether they were paired- or single-end.*

Antibodies | *Describe the antibodies used for the ChIP-seq experiments; as applicable, provide supplier name, catalog number, clone name, and lot number.*

Peak calling parameters | *Specify the command line program and parameters used for read mapping and peak calling, including the ChIP, control and index files used.*

Data quality | *Describe the methods used to ensure data quality in full detail, including how many peaks are at FDR 5% and above 5-fold enrichment.*

Software | *Describe the software used to collect and analyze the ChIP-seq data. For custom code that has been deposited into a community repository, provide accession details.*

Flow Cytometry

Plots

Confirm that:

☒ The axis labels state the marker and fluorochrome used (e.g. CD4-FITC).

☒ The axis scales are clearly visible. Include numbers along axes only for bottom left plot of group (a 'group' is an analysis of identical markers).

☐ All plots are contour plots with outliers or pseudocolor plots.

☒ A numerical value for number of cells or percentage (with statistics) is provided.

Methodology

Sample preparation | The information is provided in 'Methods' section:
Cells treated with ENMD-2076 or transfected with AURKA siRNA were harvested, washed with PBS and then fixed with cold 70% ethanol overnight at -20°C. The fixed cells were washed with PBS, suspended in 0.5 mL PBS, containing 50 µg/mL propidium

iodide (PI), 0.1 mg/mL RNase A and 0.1% Triton X-100 for 30 min at 37°C. Then, the cells were analyzed for cell cycle. Cell apoptosis was detected with FITC-Annexin V Apoptosis Detection Kit (BioLegend, San Diego, CA) following the instruction.

Instrument	Samples were analysed under a BD Accuri C6 flow cytometer. The information is provided in 'Methods' section.
Software	FACS data were analyzed with BD Accuri C6 Software (1.0.264.21). Data were further analyzed with Microsoft Excel 2013 and GraphPad Prism 6.0.
Cell population abundance	At least 10,000 events were recorded.
Gating strategy	FACS gating strategy for cell cycle analysis: First plot gating for the cells (FSC-A/SSC-A), then second plot for propidium iodide-positive single cell (FL2-A/FL2-H). FACS gating strategy for apoptosis analysis: Plot gating for the cells (FSC-A/SSC-A).

☒ Tick this box to confirm that a figure exemplifying the gating strategy is provided in the Supplementary Information.

Magnetic resonance imaging

Experimental design

Design type	*Indicate task or resting state; event-related or block design.*
Design specifications	*Specify the number of blocks, trials or experimental units per session and/or subject, and specify the length of each trial or block (if trials are blocked) and interval between trials.*
Behavioral performance measures	*State number and/or type of variables recorded (e.g. correct button press, response time) and what statistics were used to establish that the subjects were performing the task as expected (e.g. mean, range, and/or standard deviation across subjects).*

Acquisition

Imaging type(s)	*Specify: functional, structural, diffusion, perfusion.*
Field strength	*Specify in Tesla*
Sequence & imaging parameters	*Specify the pulse sequence type (gradient echo, spin echo, etc.), imaging type (EPI, spiral, etc.), field of view, matrix size, slice thickness, orientation and TE/TR/flip angle.*
Area of acquisition	*State whether a whole brain scan was used OR define the area of acquisition, describing how the region was determined.*

Diffusion MRI ☐ Used ☐ Not used

Preprocessing

Preprocessing software	*Provide detail on software version and revision number and on specific parameters (model/functions, brain extraction, segmentation, smoothing kernel size, etc.).*
Normalization	*If data were normalized/standardized, describe the approach(es): specify linear or non-linear and define image types used for transformation OR indicate that data were not normalized and explain rationale for lack of normalization.*
Normalization template	*Describe the template used for normalization/transformation, specifying subject space or group standardized space (e.g. original Talairach, MNI305, ICBM152) OR indicate that the data were not normalized.*
Noise and artifact removal	*Describe your procedure(s) for artifact and structured noise removal, specifying motion parameters, tissue signals and physiological signals (heart rate, respiration).*
Volume censoring	*Define your software and/or method and criteria for volume censoring, and state the extent of such censoring.*

Statistical modeling & inference

Model type and settings	*Specify type (mass univariate, multivariate, RSA, predictive, etc.) and describe essential details of the model at the first and second levels (e.g. fixed, random or mixed effects; drift or auto-correlation).*
Effect(s) tested	*Define precise effect in terms of the task or stimulus conditions instead of psychological concepts and indicate whether ANOVA or factorial designs were used.*

Specify type of analysis: ☐ Whole brain ☐ ROI-based ☐ Both

Statistic type for inference (See Eklund et al. 2016)	*Specify voxel-wise or cluster-wise and report all relevant parameters for cluster-wise methods.*
Correction	*Describe the type of correction and how it is obtained for multiple comparisons (e.g. FWE, FDR, permutation or Monte Carlo).*

Models & analysis

n/a	Involved in the study
☐	☐ Functional and/or effective connectivity
☐	☐ Graph analysis
☐	☐ Multivariate modeling or predictive analysis

Functional and/or effective connectivity	*Report the measures of dependence used and the model details (e.g. Pearson correlation, partial correlation, mutual information).*
Graph analysis	*Report the dependent variable and connectivity measure, specifying weighted graph or binarized graph, subject- or group-level, and the global and/or node summaries used (e.g. clustering coefficient, efficiency, etc.).*
Multivariate modeling and predictive analysis	*Specify independent variables, features extraction and dimension reduction, model, training and evaluation metrics.*
